# Traffic-related air pollution and *APOE4* can synergistically affect hippocampal volume in older women: new findings from UK Biobank

**DOI:** 10.3389/frdem.2024.1402091

**Published:** 2024-07-29

**Authors:** Vladimir A. Popov, Svetlana V. Ukraintseva, Hongzhe Duan, Anatoliy I. Yashin, Konstantin G. Arbeev

**Affiliations:** Biodemography of Aging Research Unit, Social Science Research Institute, Duke University, Durham, NC, United States

**Keywords:** hippocampal volume, neurodegeneration, air pollution, TRAP, major road, *APOE*, aging, Alzheimer's disease

## Abstract

A growing research body supports the connection between neurodegenerative disorders, including Alzheimer's disease (AD), and traffic-related air pollution (TRAP). However, the underlying mechanisms are not well understood. A deeper investigation of TRAP effects on hippocampal volume (HV), a major biomarker of neurodegeneration, may help clarify these mechanisms. Here, we explored TRAP associations with the HV in older participants of the UK Biobank (UKB), taking into account the presence of *APOE* e4 allele (*APOE4*), the strongest genetic risk factor for AD. Exposure to TRAP was approximated by the distance of the participant's main residence to the nearest major road (DNMR). The left/right HV was measured by magnetic resonance imaging (MRI) in cubic millimeters (mm^3^). Analysis of variance (ANOVA), Welch test, and regression were used to examine statistical significance. We found significant interactions between DNMR and *APOE4* that influenced HV. Specifically, DNMR <50m (equivalent of a chronically high exposure to TRAP), and carrying *APOE4* were synergistically associated with a significant (*P* = 0.01) reduction in the right HV by about 2.5% in women aged 60–75 years (results for men didn't reach a statistical significance). Results of our study suggest that TRAP and *APOE4* jointly promote neurodegeneration in women. Living farther from major roads may help reduce the risks of neurodegenerative disorders, including AD, in female *APOE4* carriers.

## 1 Introduction

A growing body of research points to a connection between exposure to air pollution and neurodegenerative disorders, including Alzheimer's disease (AD), though mechanisms are not fully understood (Tham and Schikowski, 2021; Parra et al., 2022; Finch, 2023; Franz et al., 2023; Yuan et al., 2023). Various pollutants are present in the air, and some may pose risks to human health. For example, inhalable particulate matter (PM) and nitrogen dioxide (NO_2_) have been intensively studied in this regard (Akimoto, 2003; Craig et al., 2008; Dominski et al., 2021). A recent study of UKB (UK Biobank, 2023) data found that higher exposure to PM_2.5_ (median particle with diameter ≤ 2.5 μm) and NO_2_ was associated with multimorbidity in a dose-dependent manner (Ronaldson et al., 2022). The PM, NO_2_, and volatile organic compounds (VOCs) are common components of the traffic-related air pollution (TRAP). These and other types of air pollution (such as ozone, sulfur oxides, carbon monoxide, and lead), might be harmful to the central nervous system (CNS) and promote neuroinflammation and neurodegeneration (Hogan et al., 2015; Calderón-Garcidueñas et al., 2016a; Cheng et al., 2016; Spangenberg and Green, 2017; Costa et al., 2020). A review of epidemiological and experimental studies of the role of PM in neurodegeneration emphasized a link between chronic exposure to PM and onsets of cognitive deficits, dementia, and AD (You et al., 2022). A meta-analysis of 14 studies concluded that PM_2.5_ is a risk factor for dementia, with more limited support for nitrogen oxides, though the authors stressed that these results should be interpreted with caution (Wilker et al., 2023). Higher exposure to NO_2_ itself was associated with lower cortical thickness of brain regions relevant to AD (Crous-Bou et al., 2020). Another study that used the UKB data (Li et al., 2023) reported an association between residential distance to major roads and dementia that was mediated by TRAP, mainly NO_2_.

Exposure to environmental pollutants, including TRAP, could be especially detrimental for hippocampus, a key brain structure for learning and memory, and a primary brain region affected by AD (van der Flier and Scheltens, 2009; Rao et al., 2022). Hippocampal atrophy, manifested in reduced hippocampal volume (HV), is considered one of the major biomarkers of neurodegeneration and preclinical AD pathology (Jack et al., 2018; Grober et al., 2021). It has been associated with a decline in cognitive function and progression of mild cognitive impairment (MCI) to AD (Jack et al., 2000; Henneman et al., 2009; Qu et al., 2023). It was shown that exposure to PM can create profound metabolic disturbances in hippocampus, and adversely affect HV (Park et al., 2020; Balboni et al., 2022). A study that used brain imaging and air pollution data from the UKB found an association between higher PM_2.5_ concentration and smaller left HV in adult UKB participants (Hedges et al., 2019).

Genetic factors may also influence HV. For example, carrying the *APOE* e4 allele (*APOE4*), the strongest genetic risk factor for AD, may accelerate hippocampal atrophy, along with cognitive decline (Abushakra et al., 2020). Several studies (Tohgi et al., 1997; Reiman et al., 1998; O'Dwyer et al., 2012; Saeed et al., 2021) reported that individuals with *APOE4* have markedly smaller HV, along with increased risks of AD and other dementias, compared to those without *APOE4*. The *APOE4* may also interact with exposure to air pollution, including TRAP, potentially modifying its effects on AD-related traits (Schikowski et al., 2015; Ma et al., 2023).

In this study, we used the UKB data to further explore the interactions between *APOE4* and TRAP, to better understand how the exposure to TRAP may influence HV in older adults, who carry the strongest genetic risk factor for AD.

## 2 Materials and methods

### 2.1 Data and phenotypes

This study was performed using the UKB (UK Biobank, 2023), a population-based study with extensive genetic and phenotypic data for approximately 500,000 individuals from across the UK. Data for the study were obtained (November, 2019) from the UKB database. Written informed consent was obtained by the UKB from the participants in accordance with the UK national legislation and the UKB requirements. The latest (at the time of calculations) available information on participants' withdrawal in UKB was taken into account.

In our analysis, TRAP was approximated by the participant's residence distance (in meters) to the nearest major road (DNMR). The DNMR was defined based on the local road network taken from the Ordnance Survey Meridian 2 road network 2009 with scale 1:50,000 and one meter accuracy (McGarva, 2017; Environmental Exposures Metadata and Resource 2010 UK, 2023). The median value of the DNMR was 377.4 (interquartile range: [165.9, 751.9]).

Among those subjects who had both DNMR and *APOE4* carrier status information, participants aged between 60 and 75 years, who attended the assessment center during the first imaging visit (starting January 1, 2014), were chosen. The *APOE4* carrier status was approximated by carrying C allele of the SNP rs429358. The left and right HV were measured in cubic millimeters (mm^3^), and respective information was obtained from the UKB data-fields 25019 and 25020. To normalize for head size, these measurements were multiplied by the head size scaling factor obtained from the UKB data-field 25,000 (Smith et al., 2022; Supplementary material, MRI measurements).

The analytic sample (Table 1) was divided into one factor and two factor groups, as follows:

**Table 1:** UKB sample used for analysis.

**Group/subjects**	**Female, age 60–75**	**Male, age 60–75**
DNMR	661	584
noDNMR	9,968	9,102
APOE4	2,969	2,627
noAPOE4	7,660	7,059
DNMR APOE4	199	167
DNMR noAPOE4	462	417
noDNMR APOE4	2,770	2,460
noDNMR noAPOE4	7,198	6,642
All	10,629	9,686

G1. One factor groups

DNMR group consists of subjects with residential proximity to the nearest major road <50m, noDNMR group consists of subjects with residential proximity to the nearest major road more than 50m, APOE4 group consists of *APOE4* carriers, noAPOE4 group consists of *APOE4* non-carriers.

G2. Two factor groups

DNMR_APOE4 group contains subjects from both DNMR and APOE4 groups, DNMR_noAPOE4 group contains subjects from both DNMR and noAPOE4 groups, noDNMR_APOE4 group contains subjects from both noDNMR and APOE4 groups, noDNMR_noAPOE4 group contains subjects from both noDNMR and noAPOE4 groups.

The study sample contained participants having DNMR and *APOE4* carrier status data, who attended the assessment center during the first imaging visit (between January 1, 2014 and October 31, 2019) at age 60–75 years. It, thus, only included individuals, who were at risk for the late-onset but not the early onset AD.

### 2.2 Analytic approach

Analysis of variance (ANOVA), the Tukey's test, and the Welch test (Welch, 1947; Chambers et al., 1992; Yandell, 1997) were utilized. We considered three sets of regression models Set1 = HV~*Age*,*dnmr* (8 models), Set2 = HV~*Age*,*snp* (8 models), and Set3 = HV~*Age*,*snp*,*dnmr* (64 models) (Supplementary material, Analytic approach) having HV as a response variable HV = HV (mm^3^) left/right and independent variables: *dnmr* = 1 (DNMR <50), *dnmr* = 0 (DNMR ≥ 50), *snp* = 1 (*APOE4* carrier), *snp* = 0 (*APOE4* non-carrier), and age at the time attending assessment center during the first imaging visit as the *Age* variable.

The regression models were evaluated using the Akaike information criterion (AIC) (Akaike, 1973). The optimal, with respect to the minimal AIC criteria, significant results for regression model were found for the regression sets described above. Here, significant regression model means that all regression coefficients were significant (*P* < 0.05) in a specific model, non-significance means the opposite. R standard software packages (version 3.6.3), along with *glmulti* package (Calcagno, 2022), were utilized.

## 3 Results

**Figure 1:**
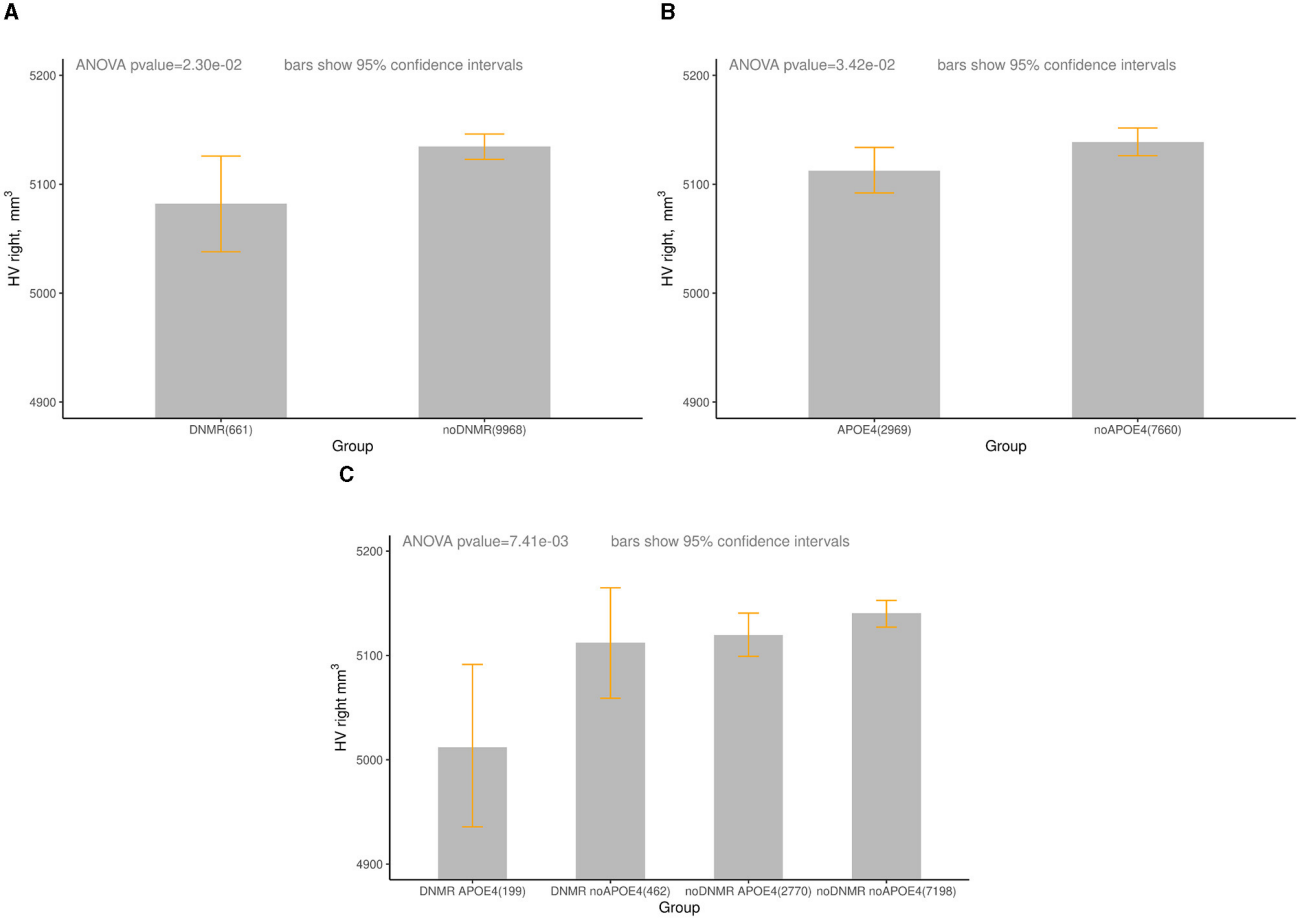
The right HV (mm^3^) in groups of women aged 60-75 years, by DNMR and APOE4 status. Age—age at the time attending the assessment center during the first imaging visit between January 1, 2014 and October 31, 2019. **(A)** UKB, HV right, DNMR <50 m, females, aged 60–75 years. DNMR (HV: mean = 5,082, 95% CI: 5,038–5,128), noDNMR (HV: mean = 5,135, 95% CI: 5,123–5,146). **(B)** UKB, HV right, APOE4, females, aged 60–75 years. APOE4 (HV: mean = 5,112, 95% CI: 5,091–5,134), no APOE4 (HV: 5,139, 95% CI: 5,126–5,151). **(C)** UKB, HV right, DNMR <50m and APOE4, females, age 60–75 years, DNMR_APOE4 (HV: mean = 5,012, 95% CI: 4,934–5,098), DNMR_noAPOE4 (HV: mean = 5,112, 95% CI: 5,059–5,161) noDNMR_APOE4 (HV: mean = 5,120, 95% CI: 5,098–5,140), noDNMR no APOE4 (HV: mean = 5,140, 95% CI: 5,127–5,154). For more detailed statistics, see Table 2.

We found significant difference in the right HV between groups DNMR and noDNMR, between groups APOE4 and noAPOE4, and between groups DNMR_APOE4 and noDNMR_noAPOE4 for females aged 60–75 years (Table 2, Figure 1). One can see that there was a 0.5% decrease in the right HV for *APOE4* carriers, a 1.0% decrease in the right HV for those with DNMR <50, and a 2.5% decrease in right HV for *APOE4* carriers with DNMR <50. Note that joint impact of DNMR and *APOE4* is larger than separate contributions of *APOE4* and DNMR (2.5% > 0.5%, 2.5% > 1.0%), or their sum (2.5% > 0.5% + 1.0%).

**Table 2:** Comparison of the right HV between groups of females aged 60–75.

**Test**	***P*-value**	**95% confidence intervals**	**HV estimate (mm^3^)**
**Females, age 60–75, HV (mm** ^3^ **) right**			
ANOVA	2.30e-02		
DNMR		[5,038, 5,128]	5,082
noDNMR		[5,123, 5,146]	5,135
DNMR – noDNMR		[−98, −7]	−53
**Females, age 60–75, HV (mm** ^3^ **) right**			
ANOVA	3.42e-02		
APOE4		[5,091, 5,134]	5,112
noAPOE4		[5,126, 5,151]	5,139
APOE4 – noAPOE4		[−51, −2]	−26
**Females, age [60–75], HV (mm** ^3^ **) right**			
Tukey	1.70e-01		
DNMR_APOE4		[4,934, 5098]	5,012
DNMR_noAPOE4		[5,059, 5,161]	5,112
DNMR_APOE4 – DNMR_noAPOE4		[−226, 25]	−100
**Females, age [60–75], HV (mm** ^3^ **) right**			
Tukey	5.37e-02		
DNMR_APOE4		[4,934, 5,098]	5,012
noDNMR_APOE4		[5,098, 5 140]	5,120
DNMR_APOE4 – noDNMR_APOE4		[−216, 1]	−108
**Females, age [60–75], HV (mm** ^3^ **) right**			
Tukey	1.04e-02		
DNMR_APOE4		[4,934, 5,098]	5,012
noDNMR_noAPOE4		[5,127, 5,154]	5,140
DNMR_APOE4 – noDNMR_noAPOE4		[−235, −22]	−128
**Females, age [60–75], HV (mm** ^3^ **) right**			
Tukey	9.94e-01		
DNMR_noAPOE4		[5,059, 5,161]	5,112
noDNMR_APOE4		[5,098, 5,140]	5,120
DNMR_noAPOE4 – noDNMR_APOE4		[−82, 67]	−7
**Females, age [60–75], HV (mm** ^3^ **) right**			
Tukey	7.36e-01		
DNMR_noAPOE4		[5,059, 5,161]	5,112
noDNMR_noAPOE4		[5,127, 5,154]	5,140
DNMR_noAPOE4 – noDNMR_noAPOE4		[−99, 43]	−28
**Females, age [60–75], HV (mm** ^3^ **) right**			
Tukey	3.66e-01		
noDNMR_APOE4		[5,098, 5,140]	5,120
noDNMR_noAPOE4		[5,127, 5,154]	5,140
noDNMR_APOE4 – noDNMR_noAPOE4		[−54, 12]	−21

In this table, differences between the following groups are considered: DNMR and noDNMR, APOE4 and noAPOE4, DNMR_APOE4 and DNMR_noAPOE4, DNMR_APOE4 and noDNMR_APOE4, DNMR_APOE4 and noDNMR_noAPOE4, DNMR_noAPOE4 and noDNMR_APOE4, DNMR_noAPOE4 and noDNMR_noAPOE4, noDNMR_APOE4 and noDNMR_noAPOE4. The sign minus between two groups denotes the difference between the means in two groups (effect size). For instance, DNMR_APOE4 - DNMR_noAPOE4 equals to the difference between the right HV mean value in the group DNMR_APOE4 and group DNMR_noAPOE4. Scientific notation “e” means that the base number is multiplied by 10 raised to the given power.

There was a 0.6% decrease in the left HV for *APOE4* carriers in women aged 60–75 years; differences between other groups were not statistically significant (Supplementary Table 1, Supplementary Figure 1). Normal aging is associated with gradual reducing of HV even without any possible adverse factors (Harman, 2001; Fotuhi et al., 2012; López-Otín et al., 2013). In our analysis, we performed the Welch test to check a possible difference in age between groups (Supplementary Tables 2, 3). We found that on average the subjects in the group DNMR were older than the subjects in the group noDNMR, which might contribute to the reduced HV in the group DNMR compared to the group noDNMR. The subjects in the group APOE4, on average, were younger than the subjects in the group noAPOE4. Note that such age difference between APOE4 and noAPOE4 groups strengthened our results because younger subjects generally tend to have bigger HV than the older ones. The subjects in the group DNMR_APOE4, on average, were the same age as the subjects in the group noDNMR_noAPOE4.

Difference in the right HV between two groups DNMR and noDNMR in the Figure 1A could be attributed to the age only, with participants in the DNMR group older than participants in the noDNMR group (Supplementary Table 3). Based on Table 3, the decrease in the right HV became more pronounced with age in women aged 60–75 years: from 0.8% at age 60 to 0.9% at age 75 for the right HV. Difference in HV between two groups APOE4 and noAPOE4 in the Figure 1B could be attributed to the age and *APOE4* carrier status, with participants being younger in the APOE4 compared those in noAPOE4 (Supplementary Table 3). Based on Table 3, the decrease in the right HV became more pronounced with age in women aged 60–75 years: from 0.6% at age 60 to 0.8% at age 75 for the right HV. When taking into account three factors (age, proximity to the nearest major road, and *APOE4* carrier status), based on Table 3, difference in the right HV gradually increased from 2.4% at age 60 to 2.8% at age 75, for *APOE4* carriers with proximity to the nearest major road <50m. The right HV decreased with age in women aged 60–75 years, losing about 27 mm^3^/year.

**Table 3:** Regression analysis, females, age 60–75.

**Model/term**	**Estimate**	**Std. error**	***P*-value**
**Best model for Set1, HV (mm** ^3^ **) right, female 60–75**			
(Intercept)	6,938.4 (mm^3^)	90.6	<1.00e-50
*Age*	−27.1 (mm^3^/year)	1.4	<1.00e-50
**Best model for Set2, HV (mm** ^3^ **) right, female 60–75**			
(Intercept)	6,945.9 (mm^3^)	90.6	<1.00e-50
*Age*	−27.1 (mm^3^/year)	1.4	<1.00e-50
*Age* ^*^ *snp*	−0.5 (mm^3^/year)	0.2	5.55e-03
**Best model for Set3, HV (mm** ^3^ **) right, female 60-75**			
(Intercept)	6,947.7 (mm^3^)	90.6	<1.00e-50
*Age*	−27.2 (mm^3^/year)	1.4	<1.00e-50
*Age* ^*^ *snp*	−0.4 (mm^3^/year)	0.2	3.25e-02
*snp* ^*^ *dnmr*	−106.2 (mm^3^)	41.5	1.06e-02
***Model 13 in Set3*** **(reference model)**			
(Intercept)	6,954.1 (mm^3^)	90.7	<1.00e-50
*Age*	−27.2 (mm^3^/year)	1.4	<1.00e-50
*snp*	−43.3 (mm^3^)	22.7	5.69e-02
*dnmr*	−32.7 (mm^3^)	12.2	7.51e-03

Response variable HV = HV (mm^3^) right, independent variables: *dnmr* = 1 (DNMR <50 meters), *dnmr* = 0 (DNMR ≥ 50 m), *snp* = 1 (APOE4 carrier), *snp* = 0 (APOE4 non-carrier), *Age*—age at the time attending assessment center during the first imaging visit between January 1, 2014 and October 31, 2019. All models in the following three sets of basic linear regression models with pairwise interactions were analyzed: Set 1 = HV~*Age, dnmr* (8 models), Set 2 = HV~*Age, snp* (8 models), and Set 3 = HV~*Age, snp, dnmr* (64 models). The optimal, with respect to the minimal AIC criterion significant model was determined for each set and shown in this table. Here, significance means that all regression coefficients were significant (P <0.05) in a specific model, non-significance means the opposite. See Supplementary Tables 6–8 for more detailed information about regression analysis.

For males aged 60–75 years differences in the left/right HV between studied groups (Supplementary Tables 4, 5) were not statistically significant. For males aged 60–75 years, regression analysis found that for all regression sets Set1 = HV~*Age*,*dnmr* (8 models), Set2 = HV~*Age*,*snp* (8 models), Set3 = HV~*Age*,*snp*,*dnmr* (64 models) the best (with respect to the minimal AIC criterion) models depend only on *Age* variable (Supplementary Tables 6–8).

Comparison of the regression model with interactive term with the reference model, i.e., the regression with main additive effects (Table 3, model 13 in Set3: HV~*Age*,*snp*,*dnmr*) allows estimating deviation from the reference model, which was: deviation = 106.2 + 0.4^*^Age-(43.3 + 32.7) = 54.2, for *Age* = 60 and deviation = 60.2, for *Age* = 75, that is, deviation gradually increases with age. This observation reasonably supported synergy (Roell et al., 2017) in the interaction between DNMR and APOE e4 status with respect to HV decrease.

## 4 Discussion

Our study, using the UKB data, found that female *APOE4* carriers aged 60–75 years, who live <50 meters from a major road, had the right HV that was significantly smaller (by about 2.5%) than the HV of the same age women without these conditions. We also showed for the first time that exposure to TRAP (approximated by closeness of participant's main residence to major roads), and carrying the *APOE4*, synergistically affected HV in women. These findings imply that living farther from major roads may be especially beneficial to older female *APOE4* carriers and could help reduce their risks of neurodegenerative disorders, including AD. In our study, the right HV also decreased with age in women aged 60–75 years, losing, on average, about 27 mm^3^/year. This is in agreement with an earlier report of the HV change with age by the UKB (Nobis et al., 2019).

Results of our study are broadly in line with earlier research that demonstrated that exposure to air pollution (especially to PM_2.5_) is associated with smaller brain/hippocampal volume (Wilker et al., 2015; Hedges et al., 2019; Balboni et al., 2022). Several studies investigated the role of air pollution in dementia and cognitive decline, including in *APOE4* carriers. Chen et al. (2017) reported a modest increase in hazard ratio (1.07 [95% CI: 1.06–1.08]) of dementia in people living <50 meters from a major road. Higher PM_2.5_ exposure was linked to worse cognitive function in *APOE4* carriers, but not in non-carriers (Franz et al., 2023). A paper found that associations of PM_2.5_, PM_10_, and NO_2_ with cognitive function were more pronounced in female *APOE4* carriers (Schikowski et al., 2015). Female *APOE4* carriers were also more at risk for air pollution-induced metabolic alterations in hippocampus and cognitive deficits (Calderón-Garcidueñas et al., 2015, 2016b). Another research that used the Women's Health Initiative Memory Study (WHIMS) data found that exposure to a high level of PM_2.5_ preceded onset of cognitive impairment in older women, and this relationship varied by *APOE* genotype, with the largest adverse effect seen in e4/e4 carriers (Cacciottolo et al., 2017). The authors suggested that exposure to PM in the air may accelerate neurodegeneration through various pathways, amyloidogenic, as well as independent of amyloid deposits. A more recent study tested the interaction between *APOE* genotypes and air pollution and found that the long-term exposure to ambient air pollution was associated with a more rapid cognitive decline in *APOE4* carriers (Kulick et al., 2020). Some studies, however, did not find significant interactions between the air pollution and *APOE4*. For example, a case-control study in northern Taiwan found no differences in susceptibility to air pollution-associated dementia between *APOE* genotypes (Wu et al., 2015).

One should note that DNMR, which was used as an explanatory variable in our analysis, is an indicator of aggregated exposure to various road-related pollutants, not only to those found in car exhaust fumes. Some of these pollutants may also be potentially relevant to AD pathology. E.g., the higher intensity traffic was associated with the higher concentration of airborne fungi in urban air environments. Examples include Alternaria and Cladosporium species which may cause infection and inflammation, potentially contributing to neurodegeneration (Alonso et al., 2017; Phuna and Madhavan, 2022; Muafa et al., 2024). The role of exposure to airborne fungi in AD pathology deserves separate investigation, especially in the light of our recent findings suggesting that the impact of recurrent fungal infections on AD risk can be larger than that of other types of infections, including bacterial and viral ones (Ukraintseva et al., 2023). Other road-related pollutants, such as noise (The Lancet Regional Health-Europe, 2023), light pollution (Chepesiuk, 2009; Wyse et al., 2011; Aubrecht et al., 2013), and electromagnetic fields (Ahlbom and Feychting, 2003; Kivrak et al., 2017) might also be relevant to health risks. For instance, noise is currently considered a health problem for citizens of the European Union (European Commission, 2023).

We recognize several study limitations. Since only individuals aged 60–75, who have HV measures, were included in the analysis, the sample size in this study was substantially reduced compared to the total UK Biobank sample. Also, different head-size correction (normalization) strategies might yield various volumetric results across studies (Arndt et al., 1991; Mathalon et al., 1993; Goldstein et al., 1999; Seidman et al., 1999; Sanfilipo et al., 2004; Barnes et al., 2010; O'Brien et al., 2011; Voevodskaya et al., 2014). Also, in our study we evaluated regression models using the Akaike information criterion. One should note that there is no universal procedure by which one can determine the “best model”. We applied the AIC approach calculating goodness-of-fit and model variability in order to select the most parsimonious regression model (Burnham and Anderson, 2002; Anderson, 2008; Burnham et al., 2011). Another potential limitation could be that the formal statistical association evaluated from regression analysis may not imply actual causality, which should be further studied using causal inference approaches. Finally, the UK Biobank is volunteer-based study, and so it may not represent general population, therefore, results obtained using this sample should not be extrapolated to the entire UK population, or to other populations, and need further confirmation in additional research.

## 5 Conclusion

In summary, this study found that the interaction between *APOE4* carrier status and chronic exposure to TRAP (approximated by the closeness of a participant's main residence to a major road) is associated with a significant reduction in hippocampal volume (HV) in female participants of the UK Biobank aged 60–75 years. The results for males didn't reach statistical significance. Our findings suggest that traffic-related air pollution and genetic risk factors for AD (specifically *APOE4*) can synergistically promote neurodegeneration. Living farther from major roads could help reduce the risks of neurodegenerative disorders, including AD, in older female *APOE4* carriers.

## Data availability statement

This study used de-identified data provided by the UK Biobank (https://www.ukbiobank.ac.uk). This data is not freely available to the public but can be accessed upon approval of a data request by the UK Biobank. Specific policies governing the process to access the UK Biobank data can be found online at https://www.ukbiobank.ac.uk/enable-your-research/apply-for-access.

## Ethics statement

The studies involving human subjects were approved by the Duke University Health System Institutional Review Board in accordance with the local legislation and institutional requirements. This publication includes only secondary analyses of existing data collected by the UK Biobank and does not include identifiable human data. Written informed consent for the UK Biobank participants was obtained by the UK Biobank (data holder) in accordance with the UK national legislation and the UK Biobank requirements. The latest (at time of calculations) available information on participants' withdrawal in the UK Biobank was taken into account.

## Author contributions

VP: Conceptualization, Formal analysis, Investigation, Methodology, Software, Validation, Visualization, Writing – original draft, Writing – review & editing. SU: Conceptualization, Formal analysis, Investigation, Methodology, Project administration, Supervision, Writing – original draft, Writing – review & editing. HD: Data curation, Investigation, Validation, Writing – review & editing. AY: Investigation, Methodology, Writing – review & editing. KA: Investigation, Methodology, Writing – review & editing.

## References

[B1] Abushakra S. Porsteinsson A. P. Sabbagh M. Bracoud L. Schaerer J. Power A.. (2020). APOE ε4/ε4 homozygotes with early Alzheimer's disease show accelerated hippocampal atrophy and cortical thinning that correlates with cognitive decline. Alzheimer's Dement. 6:e12117. 10.1002/trc2.12117 33304988 PMC7716452

[B2] Ahlbom A. Feychting M. (2003). Electromagnetic radiation: environmental pollution and health. Br. Med. Bull. 68, 157–165. 10.1093/bmb/ldg030 14757715

[B3] Akaike H. (1973). “Information theory and an extension of the maximum likelihood principle,” in 2nd International Symposium on Information Theory, eds. B. N. Petrov, F. Csáki, and T. Armenia (Budapest: Akadémiai Kiadó), 267–281

[B4] Akimoto H. (2003). Global air quality and pollution. Science 302, 1716–1719. 10.1126/science.1092666 14657488

[B5] Alonso R. Pisa D. Aguado B. Carrasco L. (2017). Identification of fungal species in brain tissue from Alzheimer's disease by next-generation sequencing. J. Alzheimer's Dis. 58, 55–67. 10.3233/JAD-170058 28387676

[B6] Anderson D. R. (2008). Model Based Inference in the Life Sciences: A Primer on Evidence. New York: Springer.

[B7] Arndt S. Cohen G. Alliger R. J. Swayze V. W. Andreasen N. C. (1991). Problems with ratio and proportion measures of imaged cerebral structures. Psychiatry Res. 40, 79–89. 10.1016/0925-4927(91)90031-K 1946842

[B8] Aubrecht T. G. Weil Z. M. Magalang U. J. Nelson R. J. (2013). Dim light at night interacts with intermittent hypoxia to alter cognitive and affective responses. Am. J. Physiol. Regul. Integr. Comp. Physiol. 305, R78–R86. 10.1152/ajpregu.00100.2013 23657638 PMC3727029

[B9] Balboni E. Filippini T. Crous-Bou M. Guxens M. Erickson L. D. Vinceti M. (2022). The association between air pollutants and hippocampal volume from magnetic resonance imaging: a systematic review and meta-analysis. Environm. Res. 204:111976. 10.1016/j.envres.2021.111976 34478724

[B10] Barnes J. Ridgway G. R. Bartlett J. Henley S. M. Lehmann M. Hobbs N.. (2010). Head size, age and gender adjustment in MRI studies: a necessary nuisance? Neuroimage 53, 1244–1255. 10.1016/j.neuroimage.2010.06.025 20600995

[B11] Burnham K. P. Anderson D. R. (2002). Model Selection and Multimodel Inference: A Practical Information-Theoretic Approach (2nd ed). New York, NY: Springer.

[B12] Burnham K. P. Anderson D. R. Huyvaert K. P. (2011). AIC model selection and multimodel inference in behavioral ecology: some background, observations, and comparisons. Behav. Ecol. Sociobiol. 65, 23–35. 10.1007/s00265-010-1029-6

[B13] Cacciottolo M. Wang X. Driscoll I. Woodward N. Saffari A. Reyes J.. (2017). Particulate air pollutants, APOE alleles and their contributions to cognitive impairment in older women and to amyloidogenesis in experimental models. Transl. Psychiatry 7:e1022. 10.1038/tp.2016.280 28140404 PMC5299391

[B14] Calcagno V. (2022). R Package Glmulti. Available online at: https://cran.r-project.org/web/packages/glmulti/glmulti.pdf (accessed December 11, 2023).

[B15] Calderón-Garcidueñas L. Avila-Ramírez J. Calderón-Garcidueñas A. González-Heredia T. Acuña-Ayala H. Chao C. K.. (2016a). Cerebrospinal fluid biomarkers in highly exposed PM_2.5_ urbanites: the risk of alzheimer's and parkinson's diseases in young Mexico City residents. J. Alzheimer's Dis. 54, 597–613. 10.3233/JAD-160472 27567860

[B16] Calderón-Garcidueñas L. Kulesza R. J. Doty R. L. D'Angiulli A. Torres-Jardón R. (2015). Megacities air pollution problems: Mexico City Metropolitan Area critical issues on the central nervous system pediatric impact. Environ. Res. 137, 157–169. 10.1016/j.envres.2014.12.012 25543546

[B17] Calderón-Garcidueñas L. Reynoso-Robles R. Vargas-Martínez J. Gómez-Maqueo-Chew A. Pérez-Guillé B. Mukherjee P. S.. (2016b). Prefrontal white matter pathology in air pollution exposed Mexico City young urbanites and their potential impact on neurovascular unit dysfunction and the development of Alzheimer's disease. Environ. Res. 146, 404–417. 10.1016/j.envres.2015.12.031 26829765

[B18] Chambers J. M. Freeny A. Heiberger R. M. (1992). “Analysis of variance; designed experiments,” in Chapter 5 of Statistical Models in S, eds J. M. Chambers and T. J. Hastie (Pacific Grove: Wadsworth & Brooks/Cole).

[B19] Chen H. Kwong J. C. Copes R. Tu K. Villeneuve P. J. van Donkelaar A.. (2017). Living near major roads and the incidence of dementia, Parkinson's disease, and multiple sclerosis: a population-based cohort study. Lancet 389, 718–726. 10.1016/S0140-6736(16)32399-6 28063597

[B20] Cheng H. Davis D. A. Hasheminassab S. Morgan T. E. Finch C. E. (2016). Urban traffic-derived nanoparticulate matter reduces neurite outgrowth via TNFα *in vitro*. J. Neuroinflammation 13:19. 10.1186/s12974-016-0480-3 26810976 PMC4727336

[B21] Chepesiuk R. (2009). Missing the dark: health effects of light pollution. Environ. Health Perspect. 117, A20–A27. 10.1289/ehp.117-a20 19165374 PMC2627884

[B22] Costa L. G. Cole T. B. Dao K. Chang Y. C. Coburn J. Garrick J. M. (2020). Effects of air pollution on the nervous system and its possible role in neurodevelopmental and neurodegenerative disorders. Pharmacol. Ther. 210:107523. 10.1016/j.pharmthera.2020.107523 32165138 PMC7245732

[B23] Craig L. Brook J. R. Chiotti Q. Croes B. Gower S. Hedley A.. (2008). Air pollution and public health: a guidance document for risk managers. J. Toxicol. Environ. Health Part A 71, 588–698. 10.1080/15287390801997732 18569631

[B24] Crous-Bou M. Gascon M. Gispert J. D. Cirach M. Sánchez-Benavides G. Falcon C.. (2020). Impact of urban environmental exposures on cognitive performance and brain structure of healthy individuals at risk for Alzheimer's dementia. Environ. Int. 138, 105546. 10.1016/j.envint.2020.105546 32151419

[B25] Dominski F. H. Lorenzetti Branco J. H. Buonanno G. Stabile L. Gameiro da Silva M. Andrade A. (2021). Effects of air pollution on health: a mapping review of systematic reviews and meta-analyses. Environ. Res. 201, 111487. 10.1016/j.envres.2021.111487 34116013

[B26] Environmental Exposures Metadata and Resource 2010 UK (2023). Biobank. Available online at: https://www.ukbiobank.ac.uk (accessed December 11, 2023).

[B27] European Commission (2023). Environmental Noise Directive. Available online at: https://environment.ec.europa.eu/topics/noise/environmental-noise-directive_en (accessed July 17, 2024).

[B28] Finch C. E. (2023). Air pollution, dementia, and lifespan in the socio-economic gradient of aging: perspective on human aging for planning future experimental studies. Front. Aging 4:1273303. 10.3389/fragi.2023.1273303 38034419 PMC10683094

[B29] Fotuhi M. Do D. Jack C. (2012). Modifiable factors that alter the size of the hippocampus with ageing. Nat. Rev. Neurol. 8, 189–202. 10.1038/nrneurol.2012.27 22410582

[B30] Franz C. E. Gustavson D. E. Elman J. A. Fennema-Notestine C. Hagler D. J. Baraff A.. (2023). Associations between ambient air pollution and cognitive abilities from midlife to early old age: modification by APOE genotype. J. Alzheimers. Dis. 93, 193–209. 10.3233/JAD-221054 36970897 PMC10827529

[B31] Goldstein J. M. Goodman J. M. Seidman L. J. Kennedy D. N. Makris N. Lee H.. (1999). Cortical abnormalities in schizophrenia identified by structural magnetic resonance imaging. Arch. Gen. Psychiatry 56, 537–547. 10.1001/archpsyc.56.6.537 10359468

[B32] Grober E. Papp K. V. Rentz D. M. Sperling R. A. Johnson K. A. Amariglio R. E.. (2021). Neuroimaging correlates of Stages of Objective Memory Impairment (SOMI) system. Alzheimer's Dement. 13:e12224. 10.1002/dad2.12224 35005192 PMC8719429

[B33] Harman D. (2001). Aging: overview. Ann. N. Y. Acad. Sci. 928, 1–21. 10.1111/j.1749-6632.2001.tb05631.x 11795501

[B34] Hedges D. W. Erickson L. D. Kunzelman J. Brown B. L. Gale S. D. (2019). Association between exposure to air pollution and hippocampal volume in adults in the UK Biobank. Neurotoxicology 74, 108–120. 10.1016/j.neuro.2019.06.005 31220475

[B35] Henneman W. J. Sluimer J. D. Barnes J. van der Flier W. M. Sluimer I. C. Fox N. C.. (2009). Hippocampal atrophy rates in Alzheimer disease: added value over whole brain volume measures. Neurology 72, 999–1007. 10.1212/01.wnl.0000344568.09360.31 19289740 PMC2821835

[B36] Hogan M. K. Kovalycsik T. Sun Q. Rajagopalan S. Nelson R. J. (2015). Combined effects of exposure to dim light at night and fine particulate matter on C3H/HeNHsd mice. Behav. Brain Res. 294, 81–88. 10.1016/j.bbr.2015.07.033 26235330 PMC4745096

[B37] Jack C. R. Bennett D. A. Blennow K. Carrillo M. C. Dunn B. Haeberlein S. B.. (2018). NIA-AA Research Framework: Toward a biological definition of Alzheimer's disease. Alzheimer's Dement. 14, 535–562. 10.1016/j.jalz.2018.02.018 29653606 PMC5958625

[B38] Jack C. R. Petersen R. C. Xu Y. O'Brien P. C. Smith G. E. Ivnik R. J.. (2000). Rates of hippocampal atrophy correlate with change in clinical status in aging and AD. Neurology 55, 484–489. 10.1212/WNL.55.4.484 10953178 PMC2724764

[B39] Kivrak E. G. Yurt K. K. Kaplan A. A. Alkan I. Altun G. (2017). Effects of electromagnetic fields exposure on the antioxidant defense system. J. Microsc. Ultrastruct. 5, 167–176. 10.1016/j.jmau.2017.07.003 30023251 PMC6025786

[B40] Kulick E. R. Elkind M. S. V. Boehme A. K. Joyce N. R. Schupf N. Kaufman J. D.. (2020). Long-term exposure to ambient air pollution, APOE-ε4 status, and cognitive decline in a cohort of older adults in northern Manhattan. Environ. Int. 136, 105440. 10.1016/j.envint.2019.105440 31926436 PMC7024003

[B41] Li C. Gao D. Cai Y. S. Liang J. Wang Y. Pan Y.. (2023). Relationships of residential distance to major traffic roads with dementia incidence and brain structure measures: mediation role of air pollution. Health Data Sci. 3:0091. 10.34133/hds.0091 38487203 PMC10880167

[B42] López-Otín C. Blasco M. A. Partridge L. Serrano M. Kroemer G. (2013). The hallmarks of aging. Cell 153, 1194–1217. 10.1016/j.cell.2013.05.039 23746838 PMC3836174

[B43] Ma H. Li X. Zhou T. Wang M. Heianza Y. Qi L. (2023). Long-term exposure to low-level air pollution, genetic susceptibility and risk of dementia. Int. J. Epidemiol. 52, 738–748. 10.1093/ije/dyac146 35849335

[B44] Mathalon D. H. Sullivan E. V. Rawles J. M. Pfefferbaum A. (1993). Correction for head size in brain-imaging measurements. Psychiatry Res. 50, 121–139. 10.1016/0925-4927(93)90016-B 8378488

[B45] McGarva G. (2017). GB Transportation Network (1:50 000 Meridian 2). Edinburgh: University of Edinburgh. Available online at: https://datashare.ed.ac.uk/handle/10283/2560 (accessed December 11, 2023).

[B46] Muafa M. H. M. Quach Z. M. Al-Shaarani A. A. Q. A. Nafis M. M. H. Pecoraro L. (2024). The influence of car traffic on airborne fungal diversity in Tianjin, China. Mycology. 10.1080/21501203.2023.2300343 PMC11376297 39247890

[B47] Nobis L. Manohar S. G. Smith S. M. Alfaro-Almagro F. Jenkinson M. Mackay C. E.. (2019). Hippocampal volume across age: nomograms derived from over 19,700 people in UK Biobank. NeuroImage. Clinical 23:101904. 10.1016/j.nicl.2019.101904 31254939 PMC6603440

[B48] O'Brien L. M. Ziegler D. A. Deutsch C. K. Frazier J. A. Herbert M. R. Locascio J. J. (2011). Statistical adjustments for brain size in volumetric neuroimaging studies: some practical implications in methods. Psychiatry Res. 193, 113–122. 10.1016/j.pscychresns.2011.01.007 21684724 PMC3510982

[B49] O'Dwyer L. Lamberton F. Matura S. Tanner C. Scheibe M. Miller J.. (2012). Reduced hippocampal volume in healthy young ApoE4 carriers: an MRI study. PLoS ONE 7:e48895. 10.1371/journal.pone.0048895 23152815 PMC3494711

[B50] Park S. J. Lee J. Lee S. Lim S. Noh J. Cho S. Y.. (2020). Exposure of ultrafine particulate matter causes glutathione redox imbalance in the hippocampus: a neurometabolic susceptibility to Alzheimer's pathology. Sci. Total Environ. 718:137267. 10.1016/j.scitotenv.2020.137267 32088476

[B51] Parra K. L. Alexander G. E. Raichlen D. A. Klimentidis Y. C. Furlong M. A. (2022). Exposure to air pollution and risk of incident dementia in the UK Biobank. Environ. Res. 209, 112895. 10.1016/j.envres.2022.112895 35149105 PMC8976829

[B52] Phuna Z. X. Madhavan P. (2022). A closer look at the mycobiome in Alzheimer's disease: Fungal species, pathogenesis and transmission. Eur. J. Neurosci. 55, 1291–1321. 10.1111/ejn.15599 35048439

[B53] Qu H. Ge H. Wang L. Wang W. Hu C. (2023). Volume changes of hippocampal and amygdala subfields in patients with mild cognitive impairment and Alzheimer's disease. Acta Neurol. Belg., 123,1381–1393. 10.1007/s13760-023-02235-9 37043115

[B54] Rao Y. L. Ganaraja B. Murlimanju B. V. Joy T. Krishnamurthy A. Agrawal A. (2022). Hippocampus and its involvement in Alzheimer's disease: a review. 3 Biotech 12:55. 10.1007/s13205-022-03123-4 35116217 PMC8807768

[B55] Reiman E. M. Uecker A. Caselli R. J. Lewis S. Bandy D. de Leon M. J.. (1998). Hippocampal volumes in cognitively normal persons at genetic risk for Alzheimer's disease. Ann. Neurol. 44, 288–291. 10.1002/ana.410440226 9708558

[B56] Roell K. R. Reif D. M. Motsinger-Reif A. A. (2017). An introduction to terminology and methodology of chemical synergy-perspectives from across disciplines. Front. Pharmacol. 8:158. 10.3389/fphar.2017.00158 28473769 PMC5397413

[B57] Ronaldson A. Arias de la Torre J. Ashworth M. Hansell A. L. Hotopf M. Mudway I.. (2022). Associations between air pollution and multimorbidity in the UK Biobank: A cross-sectional study. Front. Public Health 10:1035415. 10.3389/fpubh.2022.1035415 36530697 PMC9755180

[B58] Saeed U. Desmarais P. Masellis M. (2021). The APOE ε4 variant and hippocampal atrophy in Alzheimer's disease and Lewy body dementia: a systematic review of magnetic resonance imaging studies and therapeutic relevance. Expert Rev. Neurother. 21, 851–870. 10.1080/14737175.2021.1956904 34311631

[B59] Sanfilipo M. P. Benedict R. H. Zivadinov R. Bakshi R. (2004). Correction for intracranial volume in analysis of whole brain atrophy in multiple sclerosis: the proportion vs. residual method. NeuroImage 22, 1732–1743. 10.1016/j.neuroimage.2004.03.037 15275929

[B60] Schikowski T. Vossoughi M. Vierkötter A. Schulte T. Teichert T. Sugiri D.. (2015). Association of air pollution with cognitive functions and its modification by APOE gene variants in elderly women. Environ. Res. 142, 10–16. 10.1016/j.envres.2015.06.009 26092807

[B61] Seidman L. J. Faraone S. V. Goldstein J. M. Goodman J. M. Kremen W. S. Toomey R.. (1999). Thalamic and amygdala-hippocampal volume reductions in first-degree relatives of patients with schizophrenia: an MRI-based morphometric analysis. Biol. Psychiatry 46, 941–954. 10.1016/S0006-3223(99)00075-X 10509177

[B62] Smith M. S. Alfaro-Almagro F. Miller K. L. (2022). “UK Biobank brain imaging documentation, version 1.9, December 2022,” in Wellcome Centre for Integrative Neuroimaging (WIN-FMRIB) (Oxford: Oxford University). Available online at: https://biobank.ndph.ox.ac.uk (accessed December 11, 2023).

[B63] Spangenberg E. E. Green K. N. (2017). Inflammation in Alzheimer's disease: lessons learned from microglia-depletion models. Brain Behav. Immun. 61, 1–11. 10.1016/j.bbi.2016.07.003 27395435 PMC5218993

[B64] Tham R. Schikowski T. (2021). The role of traffic-related air pollution on neurodegenerative diseases in older people: an epidemiological perspective. J. Alzheimer's Dis. 79, 949–959. 10.3233/JAD-200813 33361591

[B65] The Lancet Regional Health-Europe (2023). Noise pollution: more attention is needed. The Lancet regional health. Europe 24:100577. 10.1016/j.lanepe.2022.100577 36643665 PMC9832265

[B66] Tohgi H. Takahashi S. Kato E. Homma A. Niina R. Sasaki K.. (1997). Reduced size of right hippocampus in 39- to 80-year-old normal subjects carrying the apolipoprotein E epsilon4 allele. Neurosci. Lett. 236, 21–24. 10.1016/S0304-3940(97)00743-X 9404942

[B67] UK Biobank (2023). Available online at: https://www.ukbiobank.ac.uk (accessed December 11, 2023).

[B68] Ukraintseva S. Yashkin A. P. Akushevich I. Arbeev K. Duan H. Gorbunova G.. (2023). Associations of infections and vaccines with Alzheimer's disease point to a major role of compromised immunity rather than specific pathogen in AD. medRxiv [preprint] medRxiv 2023.12.04.23299092. 10.1101/2023.12.04.23299092 38548241 PMC11060001

[B69] van der Flier W. M. Scheltens P. (2009). Alzheimer disease: hippocampal volume loss and Alzheimer disease progression. Nat. Rev. Neurol. 5, 361–362. 10.1038/nrneurol.2009.94 19578342

[B70] Voevodskaya O. Simmons A. Nordenskjöld R. Kullberg J. Ahlström H. Lind L.. (2014). The effects of intracranial volume adjustment approaches on multiple regional MRI volumes in healthy aging and Alzheimer's disease. Front. Aging Neurosci. 6:264. 10.3389/fnagi.2014.00264 25339897 PMC4188138

[B71] Welch B. L. (1947). The generalisation of student's problems when several different population variances are involved. Biometrika 34, 28–35. 10.1093/biomet/34.1-2.28 20287819

[B72] Wilker E. H. Osman M. Weisskopf M. G. (2023). Ambient air pollution and clinical dementia: systematic review and meta-analysis. BMJ 381:e071620. 10.1136/bmj-2022-071620 37019461 PMC10498344

[B73] Wilker E. H. Preis S. R. Beiser A. S. Wolf P. A. Au R. Kloog I.. (2015). Long-term exposure to fine particulate matter, residential proximity to major roads and measures of brain structure. Stroke 46, 1161–1166. 10.1161/STROKEAHA.114.008348 25908455 PMC4414870

[B74] Wu Y. C. Lin Y. C. Yu H. L. Chen J. H. Chen T. F. Sun Y.. (2015). Association between air pollutants and dementia risk in the elderly. Alzheimer's Dement. 1, 220–228. 10.1016/j.dadm.2014.11.015 27239507 PMC4876896

[B75] Wyse C. A. Selman C. Page M. M. Coogan A. N. Hazlerigg D. G. (2011). Circadian desynchrony and metabolic dysfunction; did light pollution make us fat?. Med. Hypotheses 77, 1139–1144. 10.1016/j.mehy.2011.09.023 21983352

[B76] Yandell B. S. (1997). Practical Data Analysis for Designed Experiments. Boca Raton, FL: Chapman & Hall. 10.1007/978-1-4899-3035-4

[B77] You R. Ho Y. S. Chang R. C. (2022). The pathogenic effects of particulate matter on neurodegeneration: a review. J. Biomed. Sci. 29:15. 10.1186/s12929-022-00799-x 35189880 PMC8862284

[B78] Yuan S. Huang X. Zhang L. Ling Y. Tan S. Peng M.. (2023). Associations of air pollution with all-cause dementia, Alzheimer's disease, and vascular dementia: a prospective cohort study based on 437,932 participants from the UK biobank. Front. Neurosci. 17:1216686. 10.3389/fnins.2023.1216686 37600021 PMC10436530

