# Supplementary material for: Traffic-related air pollution and APOE4 can synergistically affect hippocampal volume in older women: new findings from UK Biobank
Source: Front Dement. 2024 Jul 29;3:1402091. doi: 10.3389/frdem.2024.1402091 (PMC11317402; doi:10.3389/frdem.2024.1402091)
Supplement: Supplementary file 1 [file Data_Sheet_1.docx]

Supplementary Material

# MRI measurements

For all brain six imaging modalities T1, rfMRI, T2_FLAIR, dMRI, SWI, tfMRI, utilized in UK Biobank, the image processing pipeline has also been used to generate many IDPs (image-derived phenotypes). T1 modality provides IDPs primarily relating to volumes of brain tissues and structures, including subcortical structures’ volumes (in particular, hippocampal volumes). In UK Biobank, the left HV (data-field=25019) and the right HV (data-field=25020) are presented as IDPs, which are derived from a T1-weighted structural image during imaging modality T1 (T1-weighted imaging). In order to normalize an IDP for head size, the T1-based “headsize scaling factor” (data-field=25000) should be used. This scaling factor is estimated when transforming from native to standard space. So, to normalize variables for head size, one should multiply raw IDP values by “headsize scaling factor” (Smith et al., 2022). Thus, for our study, we obtained the left/right HV volumes and the “headsize scaling factor” (data-field=25000) from UK Biobank. By multiplying the HV by the “headsize scaling factor”, we normalized the HV.

# Analytic approach

We considered the following three sets of regression models Set1=HV~*Age*,*dnmr;* Set2=HV~*Age*,*snp;* and Set3=HV~*Age*,*snp*,*dnmr* having HV as a response response variable HV=HV(mm^3^) left/right and independent variables: *dnmr*=1 (DNMR<50), *dnmr*=0 (DNMR>=50), *apoe4*=1 (APOE e4 carrier), *apoe4*=0 (APOE e4 non-carrier), and age at the time attending assessment center during the first imaging visit (between January 1, 2014 and October 31, 2019) as the *Age* variable:

Set1=HV~*Age*,*dnmr* (8 models: all regression models having linear variables *Age, dnmr* and their pairwise interactions)

HV = *Intercept + b_1_*Age + b_2_*dnmr + b_12_*Age*dnmr*

HV *= Intercept + b_1_*Age + b_12_*Age*dnmr*

HV *= Intercept + b_2_*dnmr + b_12_*Age*dnmr*

HV *= Intercept + b_12_*Age*dnmr*

HV *= Intercept + b_1_*Age +b_2_* dnmr*

HV *= Intercept + b_1_*Age*

HV *= Intercept + b_2_*dnmr*

HV *= Intercept,*

where *Intercept* is a constant called the bias term (or intercept term), *b_1,_ b_2,_* *b_12_* are the regression coefficients corresponding to the *Age*, *apoe4*, *Age*apoe4* terms in the regression model.

Set2=HV~*Age*,*snp* (8 models: all regression models having linear variables *Age, apoe4* and their pairwise interactions)

HV = *Intercept + b_1_*Age + b_2_*apoe4 + b_12_*Age*apoe4*

HV *= Intercept + b_1_*Age + b_12_*Age*apoe4*

HV *= Intercept + b_2_*apoe4 + b_12_*Age*apoe4*

HV *= Intercept + b_12_*Age*apoe4*

HV *= Intercept + b_1_*Age +b_2_* apoe4*

HV *= Intercept + b_1_*Age*

HV *= Intercept + b_2_*apoe4*

HV *= Intercept,*

where *Intercept* is a constant called the bias term (or intercept term), *b_1_, b_2_,* *b_12_* are the regression coefficients corresponding to the *Age*, *dnmr*, *Age*dnmr* terms in the regression model.

Set3=HV~*Age*,*snp*,*dnmr* (64 models: all regression models having linear variables *Age, dnmr, apoe4* and their pairwise interactions)

*HV ~ Intercept + b_1_*Age + b_1_*dnmr + b_1_*apoe4 + b_12_*Age*dnmr + b_13_*Age*apoe4 + b_23_*dnmr*apoe4*

*…*

HV *= Intercept,*

where *Intercept* is a constant called the bias term (or intercept term), *b_1,_ b_2,_ b_3_,* *b_12_, b_13_, b_23_* are the regression coefficients corresponding to the *Age*, *dnmr*, *apoe4*, *Age*dnmr Age*apoe4, apoe4*dnmr* terms in the regression model. Only the most complicated model and the simplest are presented in the list above without showing 62 models in between these two models.

# Supplementary Tables

**Supplementary Table 1.** Comparison of the left HV between females aged 60-75 between groups: DNMR and noDNMR, APOE4 and noAPOE4, DNMR_APOE4 and DNMR_noAPOE4, DNMR_APOE4 and noDNMR_APOE4, DNMR_APOE4 and noDNMR_noAPOE4, DNMR_noAPOE4 and noDNMR_APOE4, DNMR_noAPOE4 and noDNMR_noAPOE4, noDNMR*_*APOE4 and noDNMR*_*noAPOE4. In this table, the minus sign denotes the difference between the left HV means in two groups: for instance, DNMR – noDNMR equals to the difference between two means for the left HV in the group DNMR and group noDNMR. Scientific notation ‘e’ means that the base number is multiplied by 10 raised to the given power.

| Test | P-value | 95% Confidence Intervals | HV Estimate (mm^3^) |
| --- | --- | --- | --- |
| **Females, age 60-75, HV (mm^3^) left** |  |  |  |
| ANOVA | 1.40e-01 |  |  |
| HV, DNMR |  | [4909,5006] | 4957 |
| HV, noDNMR |  | [4980,5003] | 4991 |
| DNMR - noDNMR |  | [-80,11] | -34 |
| **Females, age 60-75, HV (mm^3^) left** |  |  |  |
| ANOVA | 9.94e-03 |  |  |
| HV, APOE4 |  | [4944,4987] | 4966 |
| HV, noAPOE4 |  | [4985,5011] | 4998 |
| APOE4 - noAPOE4 |  | [-57,-8] | -32 |
| **Females, age [60-75], HV (mm3) left** |  |  |  |
| Tukey | 7.37e-01 |  |  |
| DNMR_APOE4 |  | [4836,5001] | 4921 |
| DNMR_noAPOE4 |  | [4911,5025] | 4972 |
| DNMR_APOE4 - DNMR_noAPOE4 |  | [-177,76] | -50 |
| **Females, age [60-75], HV (mm3) left** |  |  |  |
| Tukey | 6.84e-01 |  |  |
| DNMR_APOE4 |  | [4836,5001] | 4921 |
| noDNMR_APOE4 |  | [4948,4991] | 4969 |
| DNMR_APOE4 - noDNMR_APOE4 |  | [-157,62] | -47 |
| **Females, age [60-75], HV (mm3) left** |  |  |  |
| Tukey | 2.40e-01 |  |  |
| DNMR_APOE4 |  | [4836,5001] | 4921 |
| noDNMR_noAPOE4 |  | [4986,5014] | 5000 |
| DNMR_APOE4 - noDNMR_noAPOE4 |  | [-186,29] | -78 |
| **Females, age [60-75], HV (mm3) left** |  |  |  |
| Tukey | 1.00e+00 |  |  |
| DNMR_noAPOE4 |  | [4911,5025] | 4972 |
| noDNMR_APOE4 |  | [4948,4991] | 4969 |
| DNMR_noAPOE4 - noDNMR_APOE4 |  | [-72,78] | 3 |
| F**emales, age [60-75], HV (mm3) left** |  |  |  |
| Tukey | 7.50e-01 |  |  |
| DNMR_noAPOE4 |  | [4911,5025] | 4972 |
| noDNMR_noAPOE4 |  | [4986,5014] | 5000 |
| DNMR_noAPOE4 - noDNMR_noAPOE4 |  | [-100,44] | -28 |
| **Females, age [60-75], HV (mm3) left** |  |  |  |
| Tukey | 8.13e-02 |  |  |
| noDNMR_APOE4 |  | [4948,4991] | 4969 |
| noDNMR_noAPOE4 |  | [4986,5014] | 5000 |
| noDNMR_APOE4 - noDNMR_noAPOE4 |  | [-64,2] | -31 |
|  |  |  |  |

**Supplementary Table 2.** *Age* distributions for those females aged 60-75 years who attended assessment center during the first imaging visit between January 1, 2014 and October 31, 2019. *Age* was recorded at the time of the visit. The age distributions for the groups shown in the Group column.

| Group | min | max | mean | sd | number |
| --- | --- | --- | --- | --- | --- |
| *DNMR* | 60.02 | 75.00 | 67.31 | 3.99 | 661 |
| *noDNMR* | 60.00 | 74.99 | 66.97 | 4.04 | 9968 |
| *APOE4* | 60.01 | 74.99 | 66.82 | 3.99 | 2269 |
| *noAPOE4* | 60.00 | 75.00 | 67.06 | 4.06 | 7660 |
| *DNMR APOE4* | 60.03 | 74.90 | 66.91 | 3.95 | 199 |
| *DNMR noAPOE4* | 60.02 | 75.00 | 67.48 | 4.01 | 462 |
| *noDNMR APOE4* | 60.01 | 74.99 | 66.82 | 4.00 | 2770 |
| *noDNMR noAPOE4* | 60.00 | 74.99 | 67.03 | 4.06 | 7198 |
|  |  |  |  |  |  |

**Supplementary Table 3.** Welch Two Sample t-test for two age distributions for those females aged 60-75 years who attended assessment center during the first imaging visit between January 1, 2014 and October 31, 2019. *Age* was recorded at the time of the visit. The groups that were compared shown in the Group column.

| Group | P-value | 95% Confidence interval | Estimate | stderr |
| --- | --- | --- | --- | --- |
|  |  |  |  |  |
| *DNMR* : no*DNMR*   \|  \| [-0.57,0.35] \| 58.00 : 59.10 \| 0.23 \| \| --- \| --- \| --- \| --- \| | 3.63e-02 | [0.02,0.65] | 67.31 : 66.97 | 0.16 |
| *APOE4* : no*APOE4* | 6.16e-03 | [-0.41,-0.07] | 66.82 : 67.06 | 0.09 |
| *DNMR* APOE4 : *DNMR* no*APOE4* | 9.19e-02 | [-1.23,0.09] | 66.91 : 67.48 | 0.34 |
| *DNMR* APOE4 : no*DNMR* *APOE4* | 7.40e-01 | [-0.48,0.66] | 66.91 : 66.82 | 0.29 |
| *DNMR* APOE4 : no*DNMR* no*APOE4* | 6.71e-01 | [-0.68,0.44] | 66.91 : 67.03 | 0.28 |
| *DNMR* noAPOE4 : no*DNMR* *APOE4* | 1.01e-03 | [0.27,1.05] | 67.48 : 66.82 | 0.20 |
| *DNMR* noAPOE4 : no*DNMR* no*APOE4* | 2.04e-02 | [0.07,0.82] | 67.48 : 67.03 | 0.19 |
| *noDNMR* APOE4 : no*DNMR* no*APOE4* | 1.56e-02 | [-0.39,-0.04] | 66.82 : 67.03 | 0.09 |
|  |  |  |  |  |

**Supplementary Table 4.** Comparison of the right HV between males aged 60-75 between groups: DNMR and noDNMR, APOE4 and noAPOE4, DNMR_APOE4 and DNMR_noAPOE4, DNMR_APOE4 and noDNMR_APOE4, DNMR_APOE4 and noDNMR_noAPOE4, DNMR_noAPOE4 and noDNMR_APOE4, DNMR_noAPOE4 and noDNMR_noAPOE4, noDNMR*_*APOE4 and noDNMR*_*noAPOE4. In this table, the minus sign denotes the difference between the right HV means in two groups: for instance, DNMR – noDNMR equals to the difference between two means for the right HV in the group DNMR and group noDNMR. Scientific notation ‘e’ means that the base number is multiplied by 10 raised to the given power.

| Test | P-value | 95% Confidence Intervals | HV Estimate (mm^3^) |
| --- | --- | --- | --- |
| **Males, age 60-75, HV (mm^3^) right** |  |  |  |
| ANOVA | 2.22e-01 |  |  |
| HV, DNMR |  | [4805,4899] | 4853 |
| HV, noDNMR |  | [4806,4835] | 4820 |
| DNMR - noDNMR |  | [-20,86] | 33 |
| **Males, age 60-75, HV (mm^3^) right** |  |  |  |
| ANOVA | 2.65e-01 |  |  |
| HV, APOE4 |  | [4787,4836] | 4810 |
| HV, noAPOE4 |  | [4811,4842] | 4826 |
| APOE4 - noAPOE4 |  | [-44,12] | -16 |
| **Males, age [60-75], HV (mm3) right** |  |  |  |
| Tukey | 9.99e-01 |  |  |
| DNMR_APOE4 |  | [4773,4950] | 4859 |
| DNMR_noAPOE4 |  | [4789,4904] | 4851 |
| DNMR_APOE4 - DNMR_noAPOE4 |  | [-141,157] | 8 |
| **Males, age [60-75], HV (mm3) right** |  |  |  |
| Tukey | 7.36e-01 |  |  |
| DNMR_APOE4 |  | [4773,4950] | 4859 |
| noDNMR_APOE4 |  | [4782,4832] | 4807 |
| DNMR_APOE4 - noDNMR_APOE4 |  | [-78,181] | 52 |
| **Males, age [60-75], HV (mm3) right** |  |  |  |
| Tukey | 9.04e-01 |  |  |
| DNMR_APOE4 |  | [4773,4950] | 4859 |
| noDNMR_noAPOE4 |  | [4809,4841] | 4825 |
| DNMR_APOE4 - noDNMR_noAPOE4 |  | [-93,161] | 34 |
| **Males, age [60-75], HV (mm3) right** |  |  |  |
| Tukey | 5.59e-01 |  |  |
| DNMR_noAPOE4 |  | [4789,4904] | 4851 |
| noDNMR_APOE4 |  | [4782,4832] | 4807 |
| DNMR_noAPOE4 - noDNMR_APOE4 |  | [-42,130] | 44 |
| **Males, age [60-75], HV (mm3) right** |  |  |  |
| Tukey | 8.50e-01 |  |  |
| DNMR_noAPOE4 |  | [4789,4904] | 4851 |
| noDNMR_noAPOE4 |  | [4809,4841] | 4825 |
| DNMR_noAPOE4 - noDNMR_noAPOE4 |  | [-56,108] | 26 |
| **Males, age [60-75], HV (mm3) right** |  |  |  |
| Tukey | 6.28e-01 |  |  |
| noDNMR_APOE4 |  | [4782,4832] | 4807 |
| noDNMR_noAPOE4 |  | [4809,4841] | 4825 |
| noDNMR_APOE4 - noDNMR_noAPOE4 |  | [-56,20] | -18 |
|  |  |  |  |

**Supplementary Table 5.** Comparison of the left HV between males aged 60-75 between groups: DNMR and noDNMR, APOE4 and noAPOE4, DNMR_APOE4 and DNMR_noAPOE4, DNMR_APOE4 and noDNMR_APOE4, DNMR_APOE4 and noDNMR_noAPOE4, DNMR_noAPOE4 and noDNMR_APOE4, DNMR_noAPOE4 and noDNMR_noAPOE4, noDNMR*_*APOE4 and noDNMR*_*noAPOE4. In this table, the minus sign denotes the difference between the left HV means in two groups: for instance, DNMR – noDNMR equals to the difference between two means for the left HV in the group DNMR and group noDNMR. Scientific notation ‘e’ means that the base number is multiplied by 10 raised to the given power.

| Test | P-value | 95% Confidence Intervals | HV Estimate (mm^3^) |
| --- | --- | --- | --- |
| **Males, age 60-75, HV (mm^3^) left** |  |  |  |
| ANOVA | 4.55e-01 |  |  |
| HV, DNMR |  | [4643,4733] | 4688 |
| HV, noDNMR |  | [4655,4681] | 4668 |
| DNMR - noDNMR |  | [-32,71] | 20 |
| **Males, age 60-75, HV (mm^3^) left** |  |  |  |
| ANOVA | 6.15e-01 |  |  |
| HV, APOE4 |  | [4644,4689] | 4664 |
| HV, noAPOE4 |  | [4657,4686] | 4671 |
| APOE4 - noAPOE4 |  | [-35,20] | -7 |
| **Males, age [60-75], HV (mm3) left** |  |  |  |
| Tukey | 9.65e-01 |  |  |
| DNMR_APOE4 |  | [4581,4763] | 4669 |
| DNMR_noAPOE4 |  | [4644,4751] | 4695 |
| DNMR_APOE4 - DNMR_noAPOE4 |  | [-171,118] | -27 |
| **Males, age [60-75], HV (mm3) left** |  |  |  |
| Tukey | 1.00e+00 |  |  |
| DNMR_APOE4 |  | [4581,4763] | 4669 |
| noDNMR_APOE4 |  | [4638,4688] | 4664 |
| DNMR_APOE4 - noDNMR_APOE4 |  | [-121,131] | 5 |
| **Males, age [60-75], HV (mm3) left** |  |  |  |
| Tukey | 1.00e+00 |  |  |
| DNMR_APOE4 |  | [4581,4763] | 4669 |
| noDNMR_noAPOE4 |  | [4656,4685] | 4670 |
| DNMR_APOE4 - noDNMR_noAPOE4 |  | [-125,122] | -1 |
| **Males, age [60-75], HV (mm3) left** |  |  |  |
| Tukey | 7.68e-01 |  |  |
| DNMR_noAPOE4 |  | [4644,4751] | 4695 |
| noDNMR_APOE4 |  | [4638,4688] | 4664 |
| DNMR_noAPOE4 - noDNMR_APOE4 |  | [-52,115] | 31 |
| **Males, age [60-75], HV (mm3) left** |  |  |  |
| Tukey | 8.42e-01 |  |  |
| DNMR_noAPOE4 |  | [4644,4751] | 4695 |
| noDNMR_noAPOE4 |  | [4656,4685] | 4670 |
| DNMR_noAPOE4 - noDNMR_noAPOE4 |  | [-54,105] | 26 |
| **Males, age [60-75], HV (mm3) left** |  |  |  |
| Tukey | 9.78e-01 |  |  |
| noDNMR_APOE4 |  | [4638,4688] | 4664 |
| noDNMR_noAPOE4 |  | [4656,4685] | 4670 |
| noDNMR_APOE4 - noDNMR_noAPOE4 |  | [-43,31] | -6 |
|  |  |  |  |

**Supplementary Table 6.** Regression models, females/males, aged 60-75 years. Response variable HV=HV (mm^3^) left/right, independent variables: *dnmr*=1 (DNMR<50 meters), *dnmr*=0 (DNMR>=50 meters), *Age* – age at the time attending assessment center during the first imaging visit (between January 1, 2014 and October 31, 2019). Four regression sets (8 models in each set) with linear terms and their pairwise interactions, corresponding to females/males aged 60-75 and left/right HV values, were analyzed and presented in ascending order by AIC value. Signf=1 means that all regression coefficient were significant (P-value<0.05) in a specific model, Signf=0 means the opposite. For regression model a short notation used. For instance, HV ~ 1 + *Age* + *dnmr***Age* denotes a standard regression HV = Intercept + b_1_**Age* + b_2_**dnmr* + b_12_**Age***dnmr* where Intercept is a constant called the bias term (or intercept term), b_1_, b_2_, b_12_ are the regression coefficients corresponding to the *Age*, *dnmr*, *Age***dnmr* terms in the regression model.

| Regression Model | AIC | Signf |
| --- | --- | --- |
|  |  |  |
| **Females, age 60-75, HV (mm^3^) left** |  |  |
| HV ~ 1 + *Age* | 165139.83 | 1 |
| HV ~ 1 + *Age* + *dnmr***Age* | 165140.40 | 0 |
| HV ~ 1 + *Age* + *dnmr* | 165140.53 | 0 |
| HV ~ 1 + *Age* + *dnmr* + *dnmr***Age* | 165141.59 | 0 |
| HV ~ 1 + *dnmr* + *dnmr***Age* | 165467.72 | 1 |
| HV ~ 1 + *dnmr***Age* | 165495.39 | 0 |
| HV ~ 1 + *dnmr* | 165496.50 | 0 |
| HV ~ 1 | 165496.67 | 1 |
|  |  |  |
| **Females, age 60-75, HV (mm^3^) right** |  |  |
| HV ~ 1 + *Age* + *dnmr* | 164914.31 | 0 |
| HV ~ 1 + *Age* + *dnmr***Age* | 164914.37 | 0 |
| HV ~ 1 + *Age* | 164916.07 | 1 |
| HV ~ 1 + *Age* + *dnmr* + *dnmr***Age* | 164916.27 | 0 |
| HV ~ 1 + *dnmr* + *dnmr***Age* | 165283.82 | 1 |
| HV ~ 1 + *dnmr***Age* | 165302.07 | 1 |
| HV ~ 1 + *dnmr* | 165303.43 | 1 |
| HV ~ 1 | 165306.60 | 1 |
|  |  |  |
| **Males, age 60-75, HV (mm^3^) left** |  |  |
| HV ~ 1 + *Age* | 151392.63 | 1 |
| HV ~ 1 + *Age* + *dnmr* | 151394.45 | 0 |
| HV ~ 1 + *Age* + *dnmr***Age* | 151394.46 | 0 |
| HV ~ 1 + *Age* + *dnmr* + *dnmr***Age* | 151396.43 | 0 |
| HV ~ 1 + *dnmr* + *dnmr***Age* | 151811.73 | 1 |
| HV ~ 1 | 151836.26 | 1 |
| HV ~ 1 + *dnmr* | 151837.70 | 0 |
| HV ~ 1 + *dnmr***Age* | 151838.09 | 0 |
|  |  |  |
| **Males, age 60-75, HV (mm^3^) right** |  |  |
| HV ~ 1 + *Age* | 151950.60 | 1 |
| HV ~ 1 + *Age* + *dnmr***Age* | 151951.69 | 0 |
| HV ~ 1 + *Age* + *dnmr* | 151951.77 | 0 |
| HV ~ 1 + *Age* + *dnmr* + *dnmr***Age* | 151953.15 | 0 |
| HV ~ 1 + *dnmr* + *dnmr***Age* | 152381.30 | 1 |
| HV ~ 1 | 152398.31 | 1 |
| HV ~ 1 + *dnmr* | 152398.81 | 0 |
| HV ~ 1 + *dnmr***Age* | 152399.42 | 0 |
|  |  |  |

**Supplementary Table 7.** Regression models, females/males, aged 60-75 years. Response variable HV=HV (mm^3^) left/right, independent variables: *apoe4*=1 (APOE e4 carrier), *apoe4*=0 (APOE e4 non-carrier), *Age* – age at the time attending assessment center during the first imaging visit (between January 1, 2014 and October 31, 2019). Four regression sets (8 models in each set) with linear terms and their pairwise interactions, corresponding to females/males aged 60-75 and left/right HV values, were analyzed and presented in ascending order by AIC value. Signf=1 means that all regression coefficient were significant (P-value<0.05) in a specific model, Signf=0 means the opposite. For regression model a short notation used. For instance, HV ~ 1 + *Age* + *apoe4***Age* denotes a standard regression HV = Intercept + b_1_**Age* + b_2_**apoe4* + b_12_**Age***apoe4* where Intercept is a constant called the bias term (or intercept term), b_1_, b_2_, b_12_ are the regression coefficients corresponding to the *Age*, *apoe4*, *Age***apoe4* terms in the regression model.

| Regression Model | AIC | Signf |
| --- | --- | --- |
|  |  |  |
| **Females, age 60-75, HV (mm^3^) left** |  |  |
| HV ~ 1 + *Age* + *apoe4* + *apoe4***Age* | 165128.11 | 1 |
| HV ~ 1 + *Age* + *apoe4***Age* | 165131.08 | 1 |
| HV ~ 1 + *Age* + *apoe4* | 165131.98 | 1 |
| HV ~ 1 + *Age* | 165139.83 | 1 |
| HV ~ 1 + *apoe4* + *apoe4***Age* | 165351.99 | 1 |
| HV ~ 1 + *apoe4***Age* | 165487.04 | 1 |
| HV ~ 1 + *apoe4* | 165492.03 | 1 |
| HV ~ 1 | 165496.67 | 1 |
|  |  |  |
| **Females, age 60-75, HV (mm^3^) right** |  |  |
| HV ~ 1 + *Age* + *apoe4***Age* | 164910.38 | 1 |
| HV ~ 1 + *Age* + *apoe4* | 164910.79 | 1 |
| HV ~ 1 + *Age* + *apoe4* + *apoe4***Age* | 164911.02 | 0 |
| HV ~ 1 + *Age* | 164916.07 | 1 |
| HV ~ 1 + *apoe4* + *apoe4***Age* | 165175.48 | 1 |
| HV ~ 1 + *apoe4***Age* | 165300.08 | 1 |
| HV ~ 1 + *apoe4* | 165304.11 | 1 |
| HV ~ 1 | 165306.60 | 1 |
|  |  |  |
| **Males, age 60-75, HV (mm^3^) left** |  |  |
| HV ~ 1 + *Age* | 151392.63 | 1 |
| HV ~ 1 + *Age* + *apoe4***Age* | 151393.79 | 0 |
| HV ~ 1 + *Age* + *apoe4* | 151393.89 | 0 |
| HV ~ 1 + *Age* + *apoe4* + *apoe4***Age* | 151394.99 | 0 |
| HV ~ 1 + *apoe4* + *apoe4***Age* | 151703.87 | 1 |
| HV ~ 1 | 151836.26 | 1 |
| HV ~ 1 + *apoe4***Age* | 151836.51 | 0 |
| HV ~ 1 + *apoe4* | 151838.01 | 0 |
|  |  |  |
| **Males, age 60-75, HV (mm^3^) right** |  |  |
| HV ~ 1 + *Age* + *apoe4***Age* | 151950.29 | 0 |
| HV ~ 1 + *Age* + *apoe4* | 151950.38 | 0 |
| HV ~ 1 + *Age* | 151950.60 | 1 |
| HV ~ 1 + *Age* + *apoe4* + *apoe4***Age* | 151952.10 | 0 |
| HV ~ 1 + *apoe4* + *apoe4***Age* | 152271.94 | 1 |
| HV ~ 1 + *apoe4***Age* | 152396.65 | 0 |
| HV ~ 1 | 152398.31 | 1 |
| HV ~ 1 + *apoe4* | 152399.06 | 0 |
|  |  |  |

**Supplementary Table 8.** Regression models, females/males, aged 60-75 years. Response variable HV=HV (mm^3^) left/right, independent variables: *dnmr*=1 (DNMR<50 meters), *dnmr*=0 (DNMR>=50 meters), *apoe4*=1 (APOE e4 carrier), *apoe4*=0 (APOE e4 non-carrier), *Age* – age at the time attending assessment center during the first imaging visit (between January 1, 2014 and October 31, 2019). Four regression sets 64 models in each set) with linear terms and their pairwise interactions, corresponding to females/males aged 60-75 and left/right HV values, were analyzed and presented in ascending order by AIC value. Signf=1 means that all regression coefficient were significant (P-value<0.05) in a specific model, Signf=0 means the opposite. For regression model a short notation used. For instance, HV ~ 1 + *Age* + *dnmr* + *apoe4* + *dnmr***Age* + *apoe4***dnmr* denotes a standard regression equation HV = Intercept + b_1_**Age* + b_2_**dnmr* + b_3_**apoe4* + b_12_**Age***dnmr* + b_31_**apoe4***dnmr* where Intercept is a constant called the bias term (or intercept term), b_1_, b_2_, b_3_, b_12_, b_31_ are the regression coefficients corresponding to the *Age*, *dnmr*, *apoe4*, *Age***dnmr*, *apoe4***dnmr* terms in the regression model.

| Regression Model | AIC | Signf |
| --- | --- | --- |
|  |  |  |
| **Females, age 60-75, HV (mm^3^) left** |  |  |
| HV ~ 1 + *Age* + *apoe4* + *apoe4***Age* | 165128.11 | 1 |
| HV ~ 1 + *Age* + *apoe4* + *dnmr***Age* + *apoe4***Age* | 165128.71 | 0 |
| HV ~ 1 + *Age* + *dnmr* + *apoe4* + *apoe4***Age* | 165128.84 | 0 |
| HV ~ 1 + *Age* + *apoe4* + *apoe4***Age* + *apoe4***dnmr* | 165128.88 | 0 |
| HV ~ 1 + *Age* + *dnmr* + *apoe4* + *dnmr***Age* + *apoe4***Age* | 165129.88 | 0 |
| HV ~ 1 + *Age* + *apoe4* + *dnmr***Age* + *apoe4***Age* + *apoe4***dnmr* | 165130.40 | 0 |
| HV ~ 1 + *Age* + *dnmr* + *apoe4* + *apoe4***Age* + *apoe4***dnmr* | 165130.50 | 0 |
| HV ~ 1 + *Age* + *apoe4***Age* | 165131.08 | 1 |
| HV ~ 1 + *Age* + *dnmr* + *apoe4* + *dnmr***Age* + *apoe4***Age* + *apoe4***dnmr* | 165131.45 | 0 |
| HV ~ 1 + *Age* + *dnmr***Age* + *apoe4***Age* | 165131.74 | 0 |
| HV ~ 1 + *Age* + *dnmr* + *apoe4***Age* | 165131.87 | 0 |
| HV ~ 1 + *Age* + *apoe4***Age* + *apoe4***dnmr* | 165131.91 | 0 |
| HV ~ 1 + *Age* + *apoe4* | 165131.98 | 1 |
| HV ~ 1 + *Age* + *apoe4* + *dnmr***Age* | 165132.64 | 0 |
| HV ~ 1 + *Age* + *apoe4* + *apoe4***dnmr* | 165132.74 | 0 |
| HV ~ 1 + *Age* + *dnmr* + *apoe4* | 165132.77 | 0 |
| HV ~ 1 + *Age* + *dnmr* + *dnmr***Age* + *apoe4***Age* | 165132.85 | 0 |
| HV ~ 1 + *Age* + *dnmr***Age* + *apoe4***Age* + *apoe4***dnmr* | 165133.44 | 0 |
| HV ~ 1 + *Age* + *dnmr* + *apoe4***Age* + *apoe4***dnmr* | 165133.54 | 0 |
| HV ~ 1 + *Age* + *dnmr* + *apoe4* + *dnmr***Age* | 165133.75 | 0 |
| HV ~ 1 + *Age* + *apoe4* + *dnmr***Age* + *apoe4***dnmr* | 165134.30 | 0 |
| HV ~ 1 + *Age* + *dnmr* + *apoe4* + *apoe4***dnmr* | 165134.40 | 0 |
| HV ~ 1 + *Age* + *dnmr* + *dnmr***Age* + *apoe4***Age* + *apoe4***dnmr* | 165134.43 | 0 |
| HV ~ 1 + *Age* + *dnmr* + *apoe4* + *dnmr***Age* + *apoe4***dnmr* | 165135.28 | 0 |
| HV ~ 1 + *Age* + *apoe4***dnmr* | 165138.66 | 0 |
| HV ~ 1 + *Age* | 165139.83 | 1 |
| HV ~ 1 + *Age* + *dnmr***Age* | 165140.40 | 0 |
| HV ~ 1 + *Age* + *dnmr* | 165140.53 | 0 |
| HV ~ 1 + *Age* + *dnmr***Age* + *apoe4***dnmr* | 165140.57 | 0 |
| HV ~ 1 + *Age* + *dnmr* + *apoe4***dnmr* | 165140.61 | 0 |
| HV ~ 1 + *Age* + *dnmr* + *dnmr***Age* + *apoe4***dnmr* | 165141.46 | 0 |
| HV ~ 1 + *Age* + *dnmr* + *dnmr***Age* | 165141.59 | 0 |
| HV ~ 1 + *dnmr* + *apoe4* + *dnmr***Age* + *apoe4***Age* | 165337.76 | 1 |
| HV ~ 1 + *dnmr* + *apoe4* + *dnmr***Age* + *apoe4***Age* + *apoe4***dnmr* | 165339.32 | 0 |
| HV ~ 1 + *apoe4* + *dnmr***Age* + *apoe4***Age* | 165351.15 | 0 |
| HV ~ 1 + *dnmr* + *apoe4* + *apoe4***Age* | 165351.91 | 0 |
| HV ~ 1 + *apoe4* + *apoe4***Age* | 165351.99 | 1 |
| HV ~ 1 + *apoe4* + *apoe4***Age* + *apoe4***dnmr* | 165352.78 | 0 |
| HV ~ 1 + *apoe4* + *dnmr***Age* + *apoe4***Age* + *apoe4***dnmr* | 165353.10 | 0 |
| HV ~ 1 + *dnmr* + *apoe4* + *apoe4***Age* + *apoe4***dnmr* | 165353.77 | 0 |
| HV ~ 1 + *dnmr* + *dnmr***Age* + *apoe4***Age* | 165457.83 | 1 |
| HV ~ 1 + *dnmr* + *dnmr***Age* + *apoe4***Age* + *apoe4***dnmr* | 165459.49 | 0 |
| HV ~ 1 + *dnmr* + *apoe4* + *dnmr***Age* | 165462.59 | 1 |
| HV ~ 1 + *dnmr* + *apoe4* + *dnmr***Age* + *apoe4***dnmr* | 165463.98 | 0 |
| HV ~ 1 + *dnmr* + *dnmr***Age* + *apoe4***dnmr* | 165467.65 | 0 |
| HV ~ 1 + *dnmr* + *dnmr***Age* | 165467.72 | 1 |
| HV ~ 1 + *dnmr***Age* + *apoe4***Age* | 165485.89 | 0 |
| HV ~ 1 + *dnmr* + *apoe4***Age* | 165486.98 | 0 |
| HV ~ 1 + *apoe4***Age* | 165487.04 | 1 |
| HV ~ 1 + *dnmr***Age* + *apoe4***Age* + *apoe4***dnmr* | 165487.89 | 0 |
| HV ~ 1 + *apoe4***Age* + *apoe4***dnmr* | 165488.19 | 0 |
| HV ~ 1 + *dnmr* + *apoe4***Age* + *apoe4***dnmr* | 165488.96 | 0 |
| HV ~ 1 + *apoe4* + *dnmr***Age* | 165490.84 | 0 |
| HV ~ 1 + *dnmr* + *apoe4* | 165491.94 | 0 |
| HV ~ 1 + *apoe4* | 165492.03 | 1 |
| HV ~ 1 + *apoe4* + *apoe4***dnmr* | 165492.80 | 0 |
| HV ~ 1 + *apoe4* + *dnmr***Age* + *apoe4***dnmr* | 165492.81 | 0 |
| HV ~ 1 + *dnmr* + *apoe4* + *apoe4***dnmr* | 165493.80 | 0 |
| HV ~ 1 + *dnmr***Age* | 165495.39 | 0 |
| HV ~ 1 + *apoe4***dnmr* | 165495.94 | 0 |
| HV ~ 1 + *dnmr* | 165496.50 | 0 |
| HV ~ 1 | 165496.67 | 1 |
| HV ~ 1 + *dnmr***Age* + *apoe4***dnmr* | 165496.72 | 0 |
| HV ~ 1 + *dnmr* + *apoe4***dnmr* | 165497.45 | 0 |
|  |  |  |
| **Females, age 60-75, HV (mm^3^) right** |  |  |
| HV ~ 1 + *Age* + *apoe4***Age* + *apoe4***dnmr* | 164905.84 | 1 |
| HV ~ 1 + *Age* + *apoe4* + *apoe4***dnmr* | 164906.16 | 1 |
| HV ~ 1 + *Age* + *apoe4* + *apoe4***Age* + *apoe4***dnmr* | 164906.41 | 0 |
| HV ~ 1 + *Age* + *dnmr* + *apoe4***Age* + *apoe4***dnmr* | 164907.47 | 0 |
| HV ~ 1 + *Age* + *dnmr***Age* + *apoe4***Age* + *apoe4***dnmr* | 164907.48 | 0 |
| HV ~ 1 + *Age* + *dnmr* + *apoe4* + *apoe4***dnmr* | 164907.81 | 0 |
| HV ~ 1 + *Age* + *apoe4* + *dnmr***Age* + *apoe4***dnmr* | 164907.82 | 0 |
| HV ~ 1 + *Age* + *dnmr* + *apoe4* + *apoe4***Age* + *apoe4***dnmr* | 164908.04 | 0 |
| HV ~ 1 + *Age* + *apoe4* + *dnmr***Age* + *apoe4***Age* + *apoe4***dnmr* | 164908.04 | 0 |
| HV ~ 1 + *Age* + *apoe4***dnmr* | 164908.41 | 1 |
| HV ~ 1 + *Age* + *dnmr* + *apoe4***Age* | 164908.75 | 0 |
| HV ~ 1 + *Age* + *dnmr***Age* + *apoe4***Age* | 164908.79 | 0 |
| HV ~ 1 + *Age* + *dnmr* + *apoe4* | 164909.16 | 0 |
| HV ~ 1 + *Age* + *apoe4* + *dnmr***Age* | 164909.21 | 0 |
| HV ~ 1 + *Age* + *dnmr* + *apoe4* + *apoe4***Age* | 164909.34 | 0 |
| HV ~ 1 + *Age* + *apoe4* + *dnmr***Age* + *apoe4***Age* | 164909.39 | 0 |
| HV ~ 1 + *Age* + *dnmr* + *dnmr***Age* + *apoe4***Age* + *apoe4***dnmr* | 164909.47 | 0 |
| HV ~ 1 + *Age* + *dnmr* + *apoe4* + *dnmr***Age* + *apoe4***dnmr* | 164909.81 | 0 |
| HV ~ 1 + *Age* + *dnmr* + *apoe4* + *dnmr***Age* + *apoe4***Age* + *apoe4***dnmr* | 164910.03 | 0 |
| HV ~ 1 + *Age* + *dnmr* + *apoe4***dnmr* | 164910.31 | 0 |
| HV ~ 1 + *Age* + *dnmr***Age* + *apoe4***dnmr* | 164910.31 | 0 |
| HV ~ 1 + *Age* + *apoe4***Age* | 164910.38 | 1 |
| HV ~ 1 + *Age* + *dnmr* + *dnmr***Age* + *apoe4***Age* | 164910.72 | 0 |
| HV ~ 1 + *Age* + *apoe4* | 164910.79 | 1 |
| HV ~ 1 + *Age* + *apoe4* + *apoe4***Age* | 164911.02 | 0 |
| HV ~ 1 + *Age* + *dnmr* + *apoe4* + *dnmr***Age* | 164911.14 | 0 |
| HV ~ 1 + *Age* + *dnmr* + *apoe4* + *dnmr***Age* + *apoe4***Age* | 164911.31 | 0 |
| HV ~ 1 + *Age* + *dnmr* + *dnmr***Age* + *apoe4***dnmr* | 164912.31 | 0 |
| HV ~ 1 + *Age* + *dnmr* | 164914.31 | 0 |
| HV ~ 1 + *Age* + *dnmr***Age* | 164914.37 | 0 |
| HV ~ 1 + *Age* | 164916.07 | 1 |
| HV ~ 1 + *Age* + *dnmr* + *dnmr***Age* | 164916.27 | 0 |
| HV ~ 1 + *dnmr* + *apoe4* + *dnmr***Age* + *apoe4***Age* + *apoe4***dnmr* | 165163.20 | 0 |
| HV ~ 1 + *dnmr* + *apoe4* + *dnmr***Age* + *apoe4***Age* | 165164.42 | 1 |
| HV ~ 1 + *apoe4* + *apoe4***Age* + *apoe4***dnmr* | 165171.03 | 1 |
| HV ~ 1 + *apoe4* + *dnmr***Age* + *apoe4***Age* + *apoe4***dnmr* | 165171.43 | 0 |
| HV ~ 1 + *apoe4* + *dnmr***Age* + *apoe4***Age* | 165171.53 | 1 |
| HV ~ 1 + *dnmr* + *apoe4* + *apoe4***Age* + *apoe4***dnmr* | 165171.97 | 0 |
| HV ~ 1 + *dnmr* + *apoe4* + *apoe4***Age* | 165172.41 | 1 |
| HV ~ 1 + *apoe4* + *apoe4***Age* | 165175.48 | 1 |
| HV ~ 1 + *dnmr* + *dnmr***Age* + *apoe4***Age* + *apoe4***dnmr* | 165276.28 | 0 |
| HV ~ 1 + *dnmr* + *dnmr***Age* + *apoe4***Age* | 165277.20 | 1 |
| HV ~ 1 + *dnmr* + *apoe4* + *dnmr***Age* + *apoe4***dnmr* | 165279.42 | 0 |
| HV ~ 1 + *dnmr* + *dnmr***Age* + *apoe4***dnmr* | 165280.06 | 1 |
| HV ~ 1 + *dnmr* + *apoe4* + *dnmr***Age* | 165281.07 | 1 |
| HV ~ 1 + *dnmr* + *dnmr***Age* | 165283.82 | 1 |
| HV ~ 1 + *dnmr***Age* + *apoe4***Age* | 165295.72 | 1 |
| HV ~ 1 + *dnmr***Age* + *apoe4***Age* + *apoe4***dnmr* | 165296.32 | 0 |
| HV ~ 1 + *apoe4***Age* + *apoe4***dnmr* | 165296.50 | 1 |
| HV ~ 1 + *dnmr* + *apoe4***Age* | 165297.07 | 1 |
| HV ~ 1 + *dnmr* + *apoe4***Age* + *apoe4***dnmr* | 165297.23 | 0 |
| HV ~ 1 + *apoe4* + *apoe4***dnmr* | 165299.65 | 0 |
| HV ~ 1 + *apoe4* + *dnmr***Age* | 165299.70 | 1 |
| HV ~ 1 + *apoe4* + *dnmr***Age* + *apoe4***dnmr* | 165299.76 | 0 |
| HV ~ 1 + *apoe4***dnmr* | 165299.90 | 1 |
| HV ~ 1 + *apoe4***Age* | 165300.08 | 1 |
| HV ~ 1 + *dnmr***Age* + *apoe4***dnmr* | 165300.54 | 0 |
| HV ~ 1 + *dnmr* + *apoe4* + *apoe4***dnmr* | 165300.60 | 0 |
| HV ~ 1 + *dnmr* + *apoe4* | 165301.06 | 1 |
| HV ~ 1 + *dnmr* + *apoe4***dnmr* | 165301.23 | 0 |
| HV ~ 1 + *dnmr***Age* | 165302.07 | 1 |
| HV ~ 1 + *dnmr* | 165303.43 | 1 |
| HV ~ 1 + *apoe4* | 165304.11 | 1 |
| HV ~ 1 | 165306.60 | 1 |
|  |  |  |
| **Males, age 60-75, HV (mm^3^) left** |  |  |
| HV ~ 1 + *Age* | 151392.63 | 1 |
| HV ~ 1 + *Age* + *apoe4***Age* | 151393.79 | 0 |
| HV ~ 1 + *Age* + *apoe4* | 151393.89 | 0 |
| HV ~ 1 + *Age* + *apoe4***dnmr* | 151394.35 | 0 |
| HV ~ 1 + *Age* + *dnmr* | 151394.45 | 0 |
| HV ~ 1 + *Age* + *dnmr***Age* | 151394.46 | 0 |
| HV ~ 1 + *Age* + *apoe4* + *apoe4***Age* | 151394.99 | 0 |
| HV ~ 1 + *Age* + *dnmr* + *apoe4***Age* | 151395.60 | 0 |
| HV ~ 1 + *Age* + *dnmr***Age* + *apoe4***Age* | 151395.61 | 0 |
| HV ~ 1 + *Age* + *apoe4***Age* + *apoe4***dnmr* | 151395.67 | 0 |
| HV ~ 1 + *Age* + *dnmr* + *apoe4***dnmr* | 151395.67 | 0 |
| HV ~ 1 + *Age* + *dnmr* + *apoe4* | 151395.70 | 0 |
| HV ~ 1 + *Age* + *dnmr***Age* + *apoe4***dnmr* | 151395.70 | 0 |
| HV ~ 1 + *Age* + *apoe4* + *dnmr***Age* | 151395.71 | 0 |
| HV ~ 1 + *Age* + *apoe4* + *apoe4***dnmr* | 151395.76 | 0 |
| HV ~ 1 + *Age* + *dnmr* + *dnmr***Age* | 151396.43 | 0 |
| HV ~ 1 + *Age* + *dnmr* + *apoe4* + *apoe4***Age* | 151396.81 | 0 |
| HV ~ 1 + *Age* + *apoe4* + *dnmr***Age* + *apoe4***Age* | 151396.82 | 0 |
| HV ~ 1 + *Age* + *apoe4* + *apoe4***Age* + *apoe4***dnmr* | 151396.84 | 0 |
| HV ~ 1 + *Age* + *dnmr* + *apoe4***Age* + *apoe4***dnmr* | 151397.14 | 0 |
| HV ~ 1 + *Age* + *dnmr***Age* + *apoe4***Age* + *apoe4***dnmr* | 151397.17 | 0 |
| HV ~ 1 + *Age* + *dnmr* + *apoe4* + *apoe4***dnmr* | 151397.23 | 0 |
| HV ~ 1 + *Age* + *apoe4* + *dnmr***Age* + *apoe4***dnmr* | 151397.25 | 0 |
| HV ~ 1 + *Age* + *dnmr* + *dnmr***Age* + *apoe4***Age* | 151397.57 | 0 |
| HV ~ 1 + *Age* + *dnmr* + *dnmr***Age* + *apoe4***dnmr* | 151397.62 | 0 |
| HV ~ 1 + *Age* + *dnmr* + *apoe4* + *dnmr***Age* | 151397.67 | 0 |
| HV ~ 1 + *Age* + *dnmr* + *apoe4* + *apoe4***Age* + *apoe4***dnmr* | 151398.30 | 0 |
| HV ~ 1 + *Age* + *apoe4* + *dnmr***Age* + *apoe4***Age* + *apoe4***dnmr* | 151398.32 | 0 |
| HV ~ 1 + *Age* + *dnmr* + *apoe4* + *dnmr***Age* + *apoe4***Age* | 151398.79 | 0 |
| HV ~ 1 + *Age* + *dnmr* + *dnmr***Age* + *apoe4***Age* + *apoe4***dnmr* | 151399.10 | 0 |
| HV ~ 1 + *Age* + *dnmr* + *apoe4* + *dnmr***Age* + *apoe4***dnmr* | 151399.18 | 0 |
| HV ~ 1 + *Age* + *dnmr* + *apoe4* + *dnmr***Age* + *apoe4***Age* + *apoe4***dnmr* | 151400.26 | 0 |
| HV ~ 1 + *dnmr* + *apoe4* + *dnmr***Age* + *apoe4***Age* | 151693.75 | 1 |
| HV ~ 1 + *dnmr* + *apoe4* + *dnmr***Age* + *apoe4***Age* + *apoe4***dnmr* | 151694.70 | 0 |
| HV ~ 1 + *apoe4* + *apoe4***Age* | 151703.87 | 1 |
| HV ~ 1 + *dnmr* + *apoe4* + *apoe4***Age* | 151705.62 | 0 |
| HV ~ 1 + *apoe4* + *apoe4***Age* + *apoe4***dnmr* | 151705.73 | 0 |
| HV ~ 1 + *apoe4* + *dnmr***Age* + *apoe4***Age* | 151705.80 | 0 |
| HV ~ 1 + *dnmr* + *apoe4* + *apoe4***Age* + *apoe4***dnmr* | 151707.04 | 0 |
| HV ~ 1 + *apoe4* + *dnmr***Age* + *apoe4***Age* + *apoe4***dnmr* | 151707.42 | 0 |
| HV ~ 1 + *dnmr* + *dnmr***Age* | 151811.73 | 1 |
| HV ~ 1 + *dnmr* + *dnmr***Age* + *apoe4***Age* | 151811.84 | 0 |
| HV ~ 1 + *dnmr* + *dnmr***Age* + *apoe4***dnmr* | 151812.96 | 0 |
| HV ~ 1 + *dnmr* + *apoe4* + *dnmr***Age* | 151813.36 | 0 |
| HV ~ 1 + *dnmr* + *dnmr***Age* + *apoe4***Age* + *apoe4***dnmr* | 151813.53 | 0 |
| HV ~ 1 + *dnmr* + *apoe4* + *dnmr***Age* + *apoe4***dnmr* | 151814.80 | 0 |
| HV ~ 1 | 151836.26 | 1 |
| HV ~ 1 + *apoe4***Age* | 151836.51 | 0 |
| HV ~ 1 + *dnmr* | 151837.70 | 0 |
| HV ~ 1 + *dnmr* + *apoe4***Age* | 151837.94 | 0 |
| HV ~ 1 + *apoe4* | 151838.01 | 0 |
| HV ~ 1 + *dnmr***Age* | 151838.09 | 0 |
| HV ~ 1 + *apoe4***dnmr* | 151838.26 | 0 |
| HV ~ 1 + *dnmr***Age* + *apoe4***Age* | 151838.34 | 0 |
| HV ~ 1 + *apoe4***Age* + *apoe4***dnmr* | 151838.44 | 0 |
| HV ~ 1 + *dnmr* + *apoe4* | 151839.44 | 0 |
| HV ~ 1 + *dnmr* + *apoe4***dnmr* | 151839.48 | 0 |
| HV ~ 1 + *apoe4* + *dnmr***Age* | 151839.84 | 0 |
| HV ~ 1 + *dnmr* + *apoe4***Age* + *apoe4***dnmr* | 151839.92 | 0 |
| HV ~ 1 + *apoe4* + *apoe4***dnmr* | 151840.00 | 0 |
| HV ~ 1 + *dnmr***Age* + *apoe4***dnmr* | 151840.02 | 0 |
| HV ~ 1 + *dnmr***Age* + *apoe4***Age* + *apoe4***dnmr* | 151840.33 | 0 |
| HV ~ 1 + *dnmr* + *apoe4* + *apoe4***dnmr* | 151841.32 | 0 |
| HV ~ 1 + *apoe4* + *dnmr***Age* + *apoe4***dnmr* | 151841.81 | 0 |
|  |  |  |
| **Males, age 60-75, HV (mm^3^) right** |  |  |
| HV ~ 1 + *Age* + *apoe4***Age* | 151950.29 | 0 |
| HV ~ 1 + *Age* + *apoe4* | 151950.38 | 0 |
| HV ~ 1 + *Age* | 151950.60 | 1 |
| HV ~ 1 + *Age* + *dnmr***Age* + *apoe4***Age* | 151951.36 | 0 |
| HV ~ 1 + *Age* + *apoe4* + *dnmr***Age* | 151951.44 | 0 |
| HV ~ 1 + *Age* + *dnmr* + *apoe4***Age* | 151951.44 | 0 |
| HV ~ 1 + *Age* + *dnmr* + *apoe4* | 151951.53 | 0 |
| HV ~ 1 + *Age* + *dnmr***Age* | 151951.69 | 0 |
| HV ~ 1 + *Age* + *dnmr* | 151951.77 | 0 |
| HV ~ 1 + *Age* + *apoe4***Age* + *apoe4***dnmr* | 151951.94 | 0 |
| HV ~ 1 + *Age* + *apoe4* + *apoe4***dnmr* | 151952.03 | 0 |
| HV ~ 1 + *Age* + *apoe4* + *apoe4***Age* | 151952.10 | 0 |
| HV ~ 1 + *Age* + *apoe4***dnmr* | 151952.54 | 0 |
| HV ~ 1 + *Age* + *dnmr* + *dnmr***Age* + *apoe4***Age* | 151952.86 | 0 |
| HV ~ 1 + *Age* + *dnmr* + *apoe4* + *dnmr***Age* | 151952.94 | 0 |
| HV ~ 1 + *Age* + *dnmr* + *dnmr***Age* | 151953.15 | 0 |
| HV ~ 1 + *Age* + *apoe4* + *dnmr***Age* + *apoe4***Age* | 151953.18 | 0 |
| HV ~ 1 + *Age* + *dnmr* + *apoe4* + *apoe4***Age* | 151953.27 | 0 |
| HV ~ 1 + *Age* + *dnmr***Age* + *apoe4***Age* + *apoe4***dnmr* | 151953.35 | 0 |
| HV ~ 1 + *Age* + *dnmr* + *apoe4***Age* + *apoe4***dnmr* | 151953.43 | 0 |
| HV ~ 1 + *Age* + *apoe4* + *dnmr***Age* + *apoe4***dnmr* | 151953.43 | 0 |
| HV ~ 1 + *Age* + *dnmr* + *apoe4* + *apoe4***dnmr* | 151953.51 | 0 |
| HV ~ 1 + *Age* + *dnmr***Age* + *apoe4***dnmr* | 151953.61 | 0 |
| HV ~ 1 + *Age* + *dnmr* + *apoe4***dnmr* | 151953.71 | 0 |
| HV ~ 1 + *Age* + *apoe4* + *apoe4***Age* + *apoe4***dnmr* | 151953.77 | 0 |
| HV ~ 1 + *Age* + *dnmr* + *apoe4* + *dnmr***Age* + *apoe4***Age* | 151954.67 | 0 |
| HV ~ 1 + *Age* + *dnmr* + *dnmr***Age* + *apoe4***Age* + *apoe4***dnmr* | 151954.82 | 0 |
| HV ~ 1 + *Age* + *dnmr* + *apoe4* + *dnmr***Age* + *apoe4***dnmr* | 151954.91 | 0 |
| HV ~ 1 + *Age* + *dnmr* + *dnmr***Age* + *apoe4***dnmr* | 151955.11 | 0 |
| HV ~ 1 + *Age* + *apoe4* + *dnmr***Age* + *apoe4***Age* + *apoe4***dnmr* | 151955.18 | 0 |
| HV ~ 1 + *Age* + *dnmr* + *apoe4* + *apoe4***Age* + *apoe4***dnmr* | 151955.26 | 0 |
| HV ~ 1 + *Age* + *dnmr* + *apoe4* + *dnmr***Age* + *apoe4***Age* + *apoe4***dnmr* | 151956.64 | 0 |
| HV ~ 1 + *dnmr* + *apoe4* + *dnmr***Age* + *apoe4***Age* | 152266.58 | 1 |
| HV ~ 1 + *dnmr* + *apoe4* + *dnmr***Age* + *apoe4***Age* + *apoe4***dnmr* | 152268.56 | 0 |
| HV ~ 1 + *apoe4* + *apoe4***Age* | 152271.94 | 1 |
| HV ~ 1 + *dnmr* + *apoe4* + *apoe4***Age* | 152272.96 | 0 |
| HV ~ 1 + *apoe4* + *dnmr***Age* + *apoe4***Age* | 152273.28 | 0 |
| HV ~ 1 + *apoe4* + *apoe4***Age* + *apoe4***dnmr* | 152273.62 | 0 |
| HV ~ 1 + *dnmr* + *apoe4* + *apoe4***Age* + *apoe4***dnmr* | 152274.96 | 0 |
| HV ~ 1 + *apoe4* + *dnmr***Age* + *apoe4***Age* + *apoe4***dnmr* | 152275.26 | 0 |
| HV ~ 1 + *dnmr* + *dnmr***Age* + *apoe4***Age* | 152379.45 | 1 |
| HV ~ 1 + *dnmr* + *dnmr***Age* | 152381.30 | 1 |
| HV ~ 1 + *dnmr* + *dnmr***Age* + *apoe4***Age* + *apoe4***dnmr* | 152381.36 | 0 |
| HV ~ 1 + *dnmr* + *apoe4* + *dnmr***Age* | 152381.83 | 0 |
| HV ~ 1 + *dnmr* + *dnmr***Age* + *apoe4***dnmr* | 152383.26 | 0 |
| HV ~ 1 + *dnmr* + *apoe4* + *dnmr***Age* + *apoe4***dnmr* | 152383.82 | 0 |
| HV ~ 1 + *apoe4***Age* | 152396.65 | 0 |
| HV ~ 1 + *dnmr* + *apoe4***Age* | 152397.13 | 0 |
| HV ~ 1 + *apoe4***Age* + *apoe4***dnmr* | 152397.23 | 0 |
| HV ~ 1 + *dnmr***Age* + *apoe4***Age* | 152397.74 | 0 |
| HV ~ 1 | 152398.31 | 1 |
| HV ~ 1 + *dnmr* + *apoe4***Age* + *apoe4***dnmr* | 152398.73 | 0 |
| HV ~ 1 + *dnmr* | 152398.81 | 0 |
| HV ~ 1 + *apoe4* | 152399.06 | 0 |
| HV ~ 1 + *dnmr***Age* + *apoe4***Age* + *apoe4***dnmr* | 152399.08 | 0 |
| HV ~ 1 + *dnmr***Age* | 152399.42 | 0 |
| HV ~ 1 + *dnmr* + *apoe4* | 152399.55 | 0 |
| HV ~ 1 + *apoe4***dnmr* | 152399.73 | 0 |
| HV ~ 1 + *apoe4* + *apoe4***dnmr* | 152400.02 | 0 |
| HV ~ 1 + *apoe4* + *dnmr***Age* | 152400.16 | 0 |
| HV ~ 1 + *dnmr* + *apoe4***dnmr* | 152400.79 | 0 |
| HV ~ 1 + *dnmr***Age* + *apoe4***dnmr* | 152401.32 | 0 |
| HV ~ 1 + *dnmr* + *apoe4* + *apoe4***dnmr* | 152401.36 | 0 |
| HV ~ 1 + *apoe4* + *dnmr***Age* + *apoe4***dnmr* | 152401.78 | 0 |
|  |  |  |
|  |  |  |

**Supplementary Figures**

**
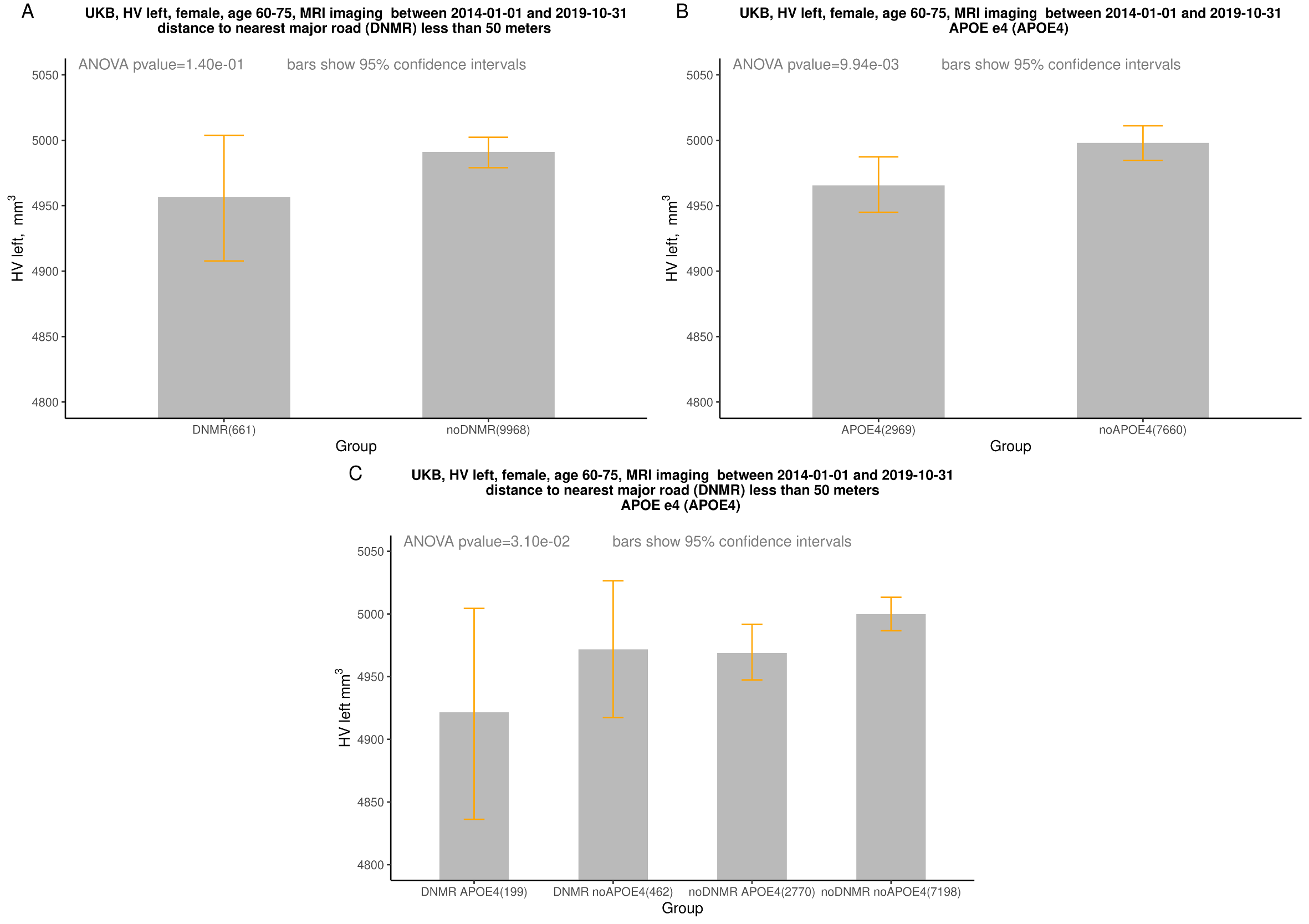
**

**Supplementaty Figure 1.** Comparison of the left HV (mm^3^) between groups of females aged 60-75 years. Age – age at the time attending assessment center during the first imaging visit between January 1, 2014 and October 31, 2019. (A) UKB, HV left, DNMR < 50 meters, females, aged 60-75 years. DNMR (HV: mean=4957, 95% CI: 4909–5006), noDNMR (HV: mean=4991, 95% CI: 4908–5003). (B) UKB, HV left, APOE4, females, aged 60-75 years. APOE4 (HV: mean=4966, 95% CI: 4944–4987), no APOE4 (HV: 4998, 95% CI: 4985–5011). (C) UKB, HV left, DNMR < 50 meters and APOE4, females, age 60-75 years, DNMR_APOE4 (HV: mean=4921, 95% CI: 4836-5001), DNMR_noAPOE4 (HV: mean=4972, 95% CI: 4911–5025) noDNMR_APOE4 (HV: mean=4969, 95% CI: 4948–4991), noDNMR noAPOE4 (HV: mean=5000, 95% CI: 4986–5014). For more detailed statistics, see Supplementary Table 1.

**References**

Smith, M. S., Alfaro-Almagro, F., and Miller, K. L. (2022). UK Biobank Brain Imaging Documentation, Version 1.9, December 2022. Wellcome Centre for Integrative Neuroimaging (WIN-FMRIB), Oxford University on behalf of UK Biobank. Available online at: https://biobank.ndph.ox.ac.uk
